# Supplementary material for: ClpC1-targeting peptide natural products differentially dysregulate the proteome of Mycobacterium tuberculosis
Source: Nat Commun. 2026 Jan 29;17:1725. doi: 10.1038/s41467-026-68423-2 (PMC12913811; doi:10.1038/s41467-026-68423-2)
Supplement: Supplementary file 1 — Supplementary Information [file 41467_2026_68423_MOESM1_ESM.pdf]

ClpC1-targeting peptide natural products differentially dysregulate the proteome of *Mycobacterium tuberculosis*

Isabel K. Barter[1,2], Max J. Bedding[1,2], Julia Leodolter[3], Joshua W. C. Maxwell[1,2], Paige M. E. Hawkins[1,2], Maxwell T. Stevens[4], Matthew B. McNeil[5], William J. Jowsey[5], Trixie Wang[4], Diana Quan[4], Sabryna Junker[3], Manuela Flórido[6], Daniel Hesselson[6], Gregory M. Cook[5], Tim Clausen[3], Warwick J. Britton[4,7 *], Mark Larance[8,*], Richard J. Payne[1,2,*].

[1] School of Chemistry, The University of Sydney, Sydney, New South Wales 2006, Australia
[2] Australian Research Council Centre of Excellence for Innovations in Peptide and Protein Science, The University of Sydney, Sydney, New South Wales 2006, Australia.
[3] Research Institute of Molecular Pathology (IMP), Dr-Bohr-Gasse 7, Vienna 1030, Austria
[4]Tuberculosis Research Program at the Centenary Institute, The University of Sydney, Sydney, New South Wales 2006, Australia.
[5] Department of Microbiology and Immunology, University of Otago, Dunedin, New Zealand.
[6]Centenary Institute and Faculty of Medicine and Health, The University of Sydney, Sydney, NSW, Australia
[7]Central Clinical School, Faculty of Medicine and Health, The University of Sydney, Sydney, New South Wales 2006, Australia
[8] Charles Perkins Centre and School of Medical Sciences, Faculty of Medicine and Health, The University of Sydney, Sydney, New South Wales 2006, Australia

*Corresponding authors: Richard J. Payne (richard.payne@sydney.edu.au), Mark Larance (mark.larance@sydney.edu.au) and Warwick Britton (warwick.britton@sydney.edu.au)

Supplementary figures, tables and information

Supplementary Figures

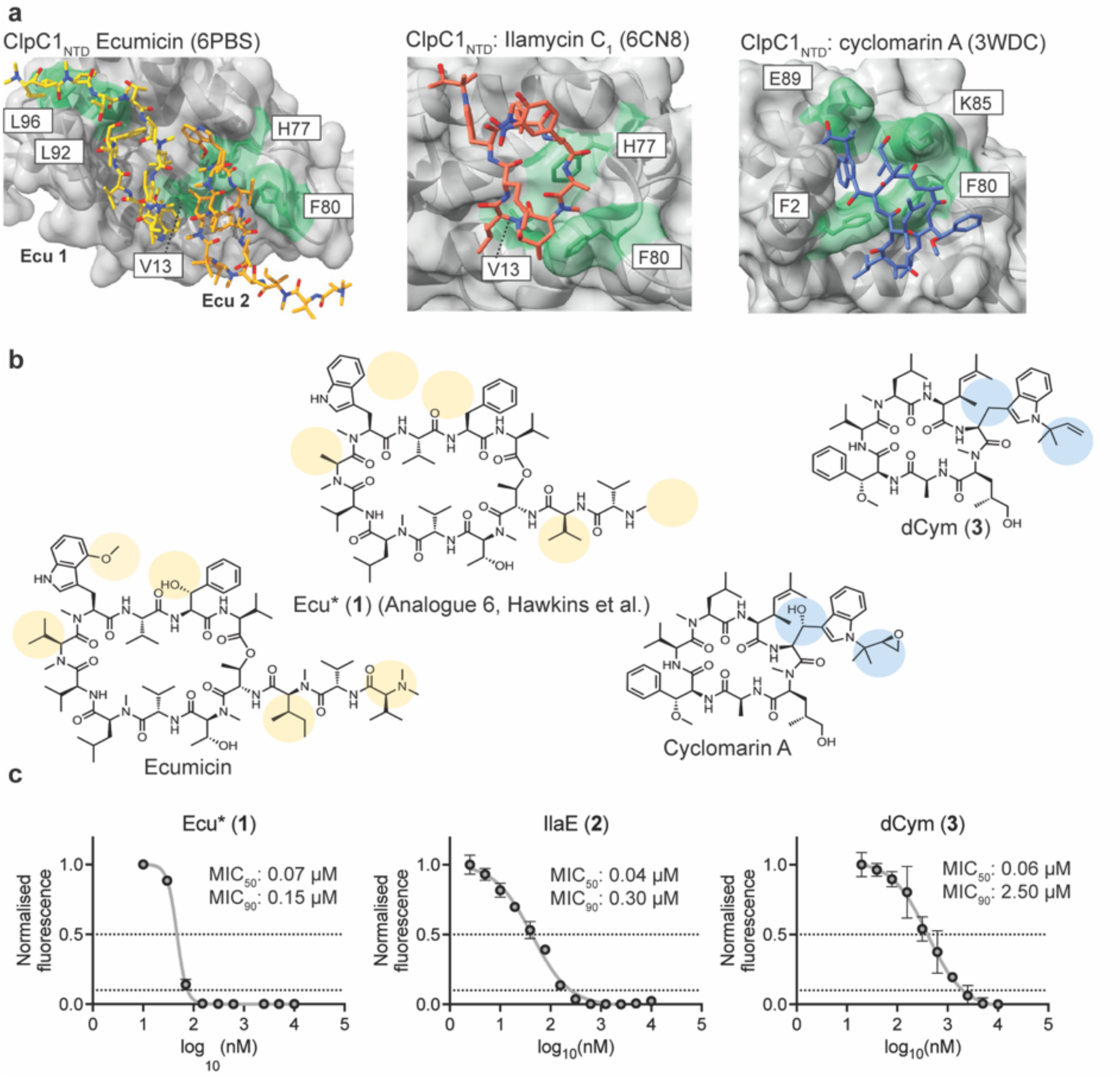

Supplementary Figure. 1 | Natural product derivatives have activity against *Mtb* H37Rv. **a** Crystal structures showing NRPs bound to ClpC1 NTD. Residues associated with resistance to each NRP labelled and highlighted in green[1-3] . **b** Chemical structures of NRP derivates used in this study. Differences between ecumicin and derivative (**1**) shown in yellow (left) and differences between cyclomarin A and desoxycyclomarin (**3**) shown in blue (right). **c** *Mtb* H37Rv growth Inhibition curves from resazurin cell viability assay. MIC_{90} and MIC_{50} were determined as the minimum inhibitory concentration to sterilise 90% or 50% of the *Mtb* H37Rv respectively**,** error bars represent SD, n = 3. Source data are provided in Source data file.

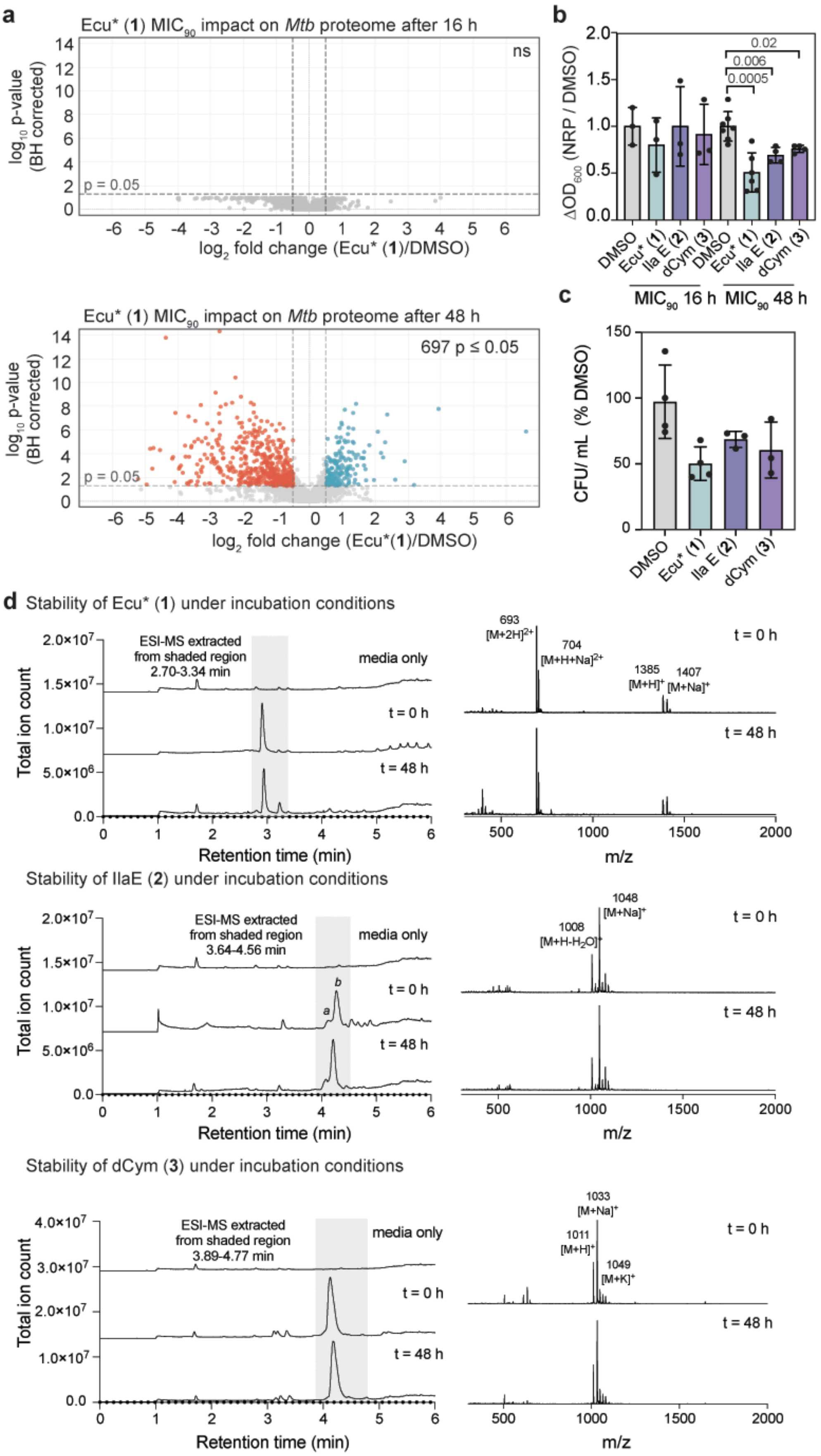
a
Ecu* (1) MIC90 impact on Mtb proteome after 16 h
ns
p = 0.05
log10 p-value (BH corrected)
log2 fold change (Ecu* (1)/DMSO)
Ecu* (1) MIC90 impact on Mtb proteome after 48 h
697 p ≤ 0.05
p = 0.05
log10 p-value (BH corrected)
log2 fold change (Ecu*(1)/DMSO)
b
ΔOD600 (NRP / DMSO)
0.02
0.006
0.0005
DMSO
Ecu* (1)
Ila E (2)
dCym (3)
MIC90 16 h
MIC90 48 h
c
CFU/ mL (% DMSO)
DMSO
Ecu* (1)
Ila E (2)
dCym (3)
d
Stability of Ecu* (1) under incubation conditions
Total ion count
ESI-MS extracted from shaded region 2.70-3.34 min
media only
t = 0 h
t = 48 h
Retention time (min)
693 [M+2H]2+
704 [M+H+Na]2+
1385 [M+H]+
1407 [M+Na]+
m/z
Stability of IlaE (2) under incubation conditions
ESI-MS extracted from shaded region 3.64-4.56 min
1048 [M+Na]+
1008 [M+H-H2O]+
Stability of dCym (3) under incubation conditions
ESI-MS extracted from shaded region 3.89-4.77 min
1033 [M+Na]+
1011 [M+H]+
1049 [M+K]+

Supplementary Figure. 2 | Determining sublethal dose of compounds for proteomic analysis. a Comparison between LC-MS/MS based proteomic analysis of *Mtb* H37Rv treated with MIC_{90} of **1** following a 16 h or a 48 h incubation. Statistical significance was calculated with a BH-adjusted one-way ANOVA, 16 h n = 3, 48 h DMSO n = 7, **1** n = 6. **b** Change in optical density at 600 nm following treatment at 16 h and 48 h. Data is presented as mean ± SEM. Statistical significance was determined using two-tailed unpaired *t*-tests, 16 h n = 3, 48 h DMSO n =7, **1** n = 6, **2** and **3** n = 4. **c** Colony forming units (CFU) *Mtb* H37Rv cell viability assay was performed to determine lethality of a treatment with the MIC_{90} of each of the NRPs **1**-**3** for 48 h. Data is presented as mean ± SEM. DMSO and **1** n = 4, **2** and **3** n = 3. **d** Stability of Ecu* (**1,** 1.0 µM**)**, IlaE (**2**, 10 µM), dCym (**3,** 1.0 µM) in Middlebrook 7H9 media containing 0.2% glycerol at 37 °C. Samples were analysed directly by UPLC-MS (0-100% MeCN in H_2O over 5 min with 0.1 vol.% FA). ***a*** and ***b*** denote *S* and *R* epimers of the 6-hydroxypiperidin-2-one motif, which readily interconvert in aqueous conditions[4,5]. Source data are provided in Source data file.

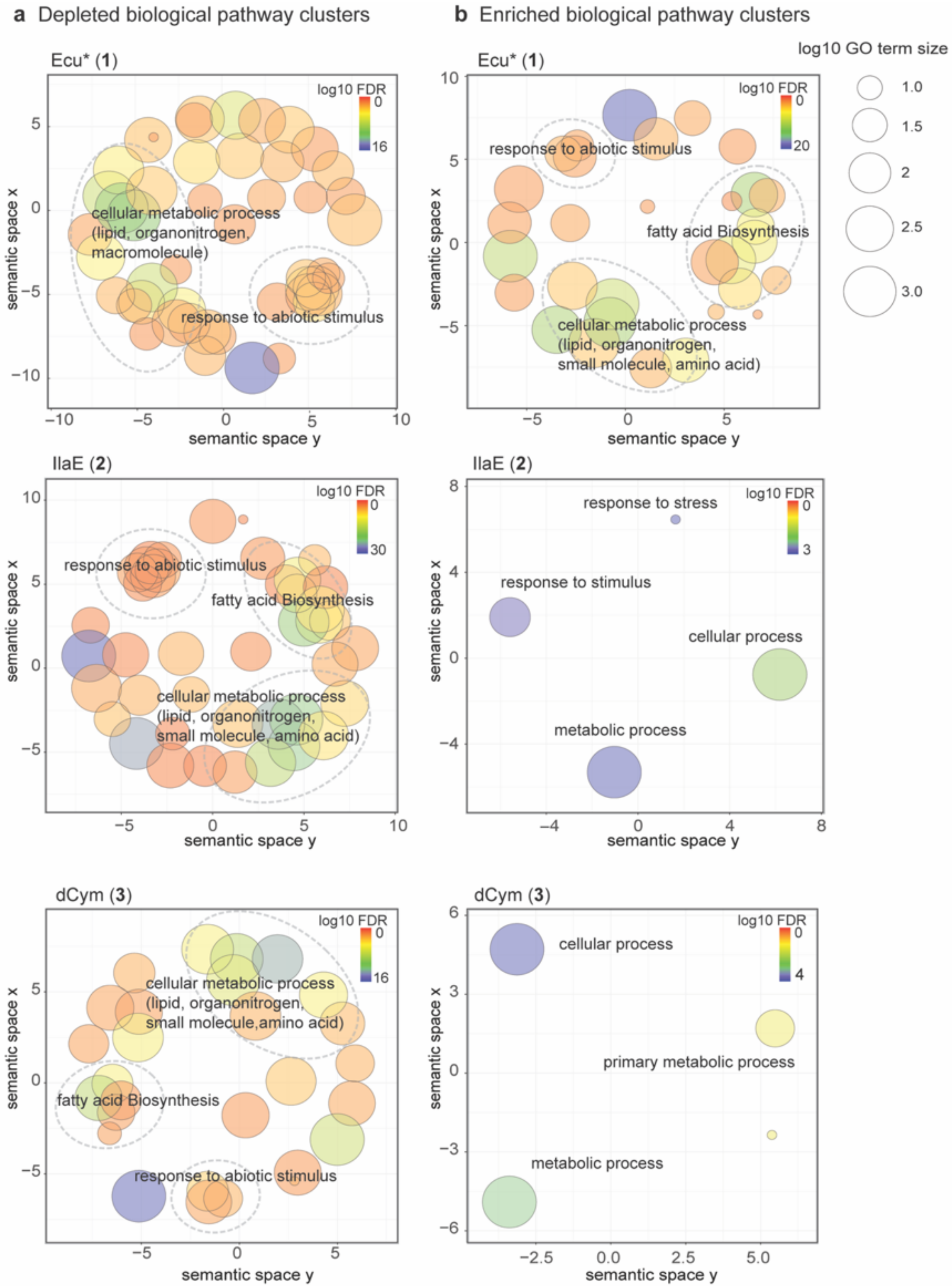
a Depleted biological pathway clusters
b Enriched biological pathway clusters
log10 GO term size
Ecu* (1)
IlaE (2)
dCym (3)
log10 FDR
semantic space x
semantic space y
cellular metabolic process (lipid, organonitrogen, macromolecule)
response to abiotic stimulus
fatty acid Biosynthesis
cellular metabolic process (lipid, organonitrogen, small molecule, amino acid)
response to stress
response to stimulus
cellular process
metabolic process
primary metabolic process

Supplementary Figure. 3 | Clustering of NRP-enriched biological process highlights key similarities and differences. Clustering of enriched biological processes from depleted protein datasets (**a**) and enriched protein datasets (**b**) for each NRP. Clustering was performed using the Revigo platform[6] and visualised with R studio. The Size of the circle's corresponds to size of Gene ontology term and colour represents the false discovery rate associated with its enrichment. Major clusters are encircled and labelled. The full list of cluster names and locations are reported in Supplementary Data 2.

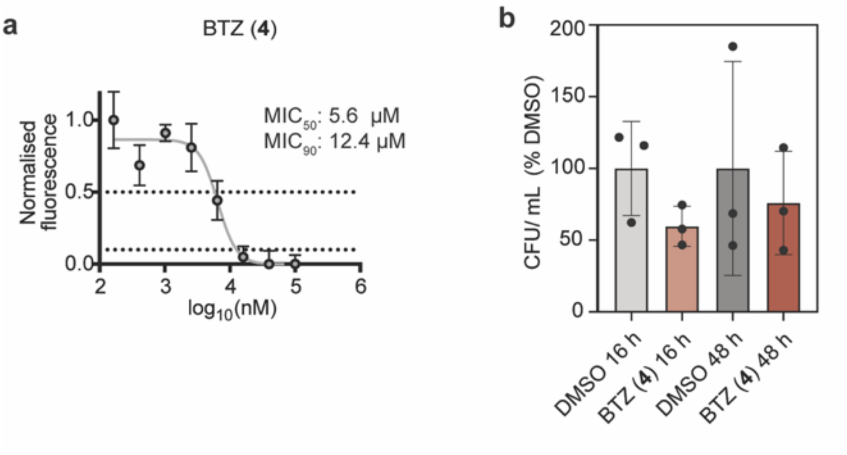

Supplementary Figure. 4 | Determining sublethal dose of BTZ (4) for proteomic analysis. a *Mtb* H37Rv growth Inhibition curve for BTZ from resazurin cell viability assay. **b** Colony forming units (CFU) *Mtb* H37Rv cell viability assay was performed to determine lethality of a treatment with the MIC_{50} of each of BTZ (**4)** for 16 h and 48 h. Data is presented as mean ± SEM, n = 3. Source data are provided in Source data file.

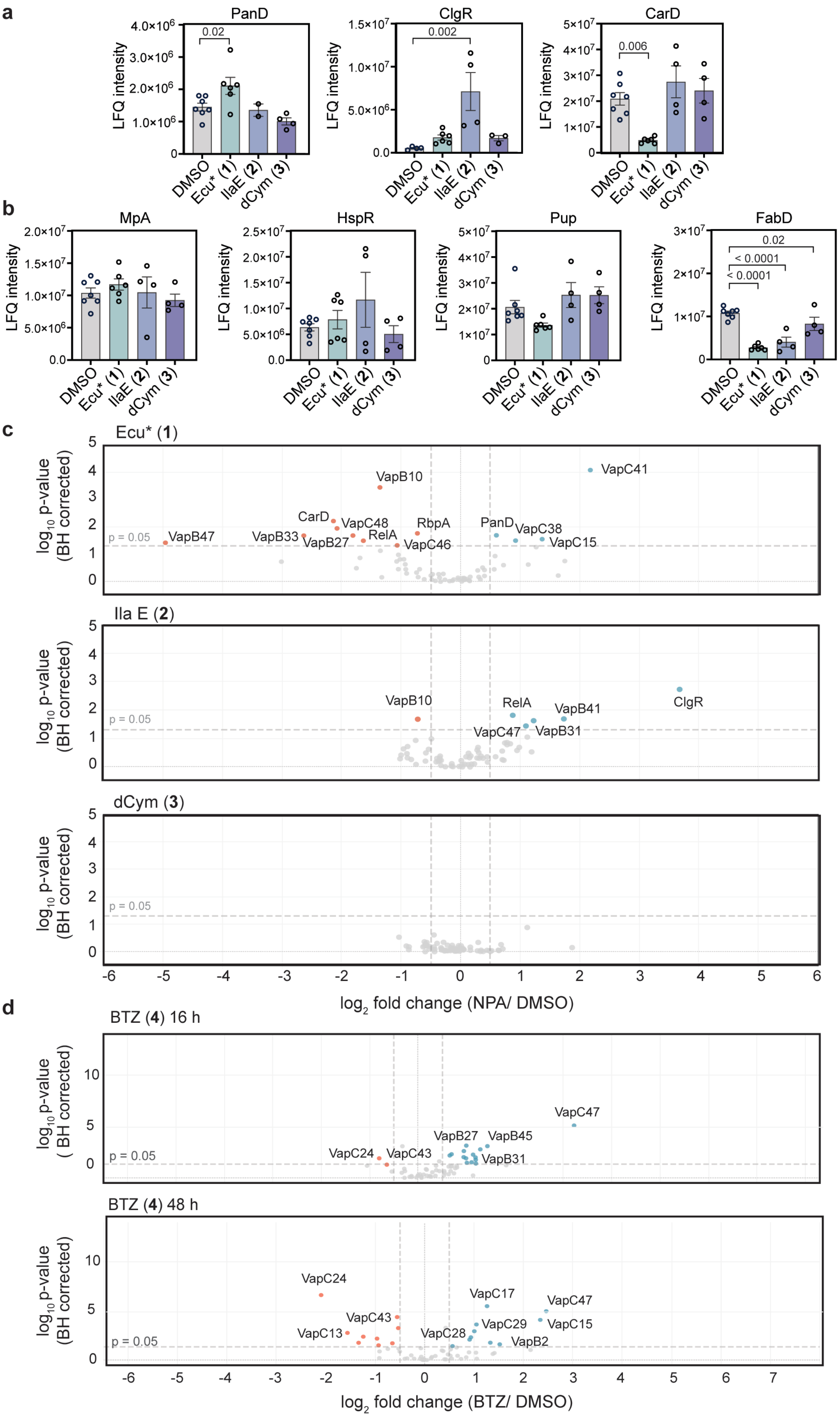
a
PanD
ClgR
CarD
LFQ intensity
0.02
0.002
0.006
DMSO
Ecu* (1)
IlaE (2)
dCym (3)
b
MpA
HspR
Pup
FabD
0.02
< 0.0001
< 0.0001
c
Ecu* (1)
Ila E (2)
dCym (3)
log10 p-value (BH corrected)
p = 0.05
VapB10
VapC41
CarD
VapC48
RbpA
PanD
VapC38
VapB47
VapB33
VapB27
RelA
VapC46
VapC15
VapB41
ClgR
VapC47
VapB31
log2 fold change (NPA/ DMSO)
d
BTZ (4) 16 h
BTZ (4) 48 h
VapC47
VapB27
VapB45
VapC24
VapC43
VapB31
VapC17
VapC29
VapC15
VapC13
VapC28
VapB2
log2 fold change (BTZ/ DMSO)

Supplementary Figure. 5| Fate of protease substrates following NRP treatment. **a** LFQ intensities for validated ClpC1 substrates. **b** LFQ intensities for validated 20S proteosome substrates. Data is presented as mean ± SEM, DMSO n = 7, **1** n = 6, **2** and **3** n = 4. Statistical analysis performed with BH-adjusted one-way ANOVA. **c, d** Volcano plots showing fold changes of validated ClpC1 substrates and suspected substrates belonging to the Rel and Vap protein families following NRP (**c**) and BTZ (**d**) treatment. DMSO n = 7, **1** n = 6, **2** and **3** n = 4. Statistical analysis performed with BH-adjusted one-way ANOVA. Proteins that became significantly depleted are labelled and shown in red. Proteins that became significantly enriched are labelled and shown in blue. Source data are provided is Supplementary Data 1 and 4.

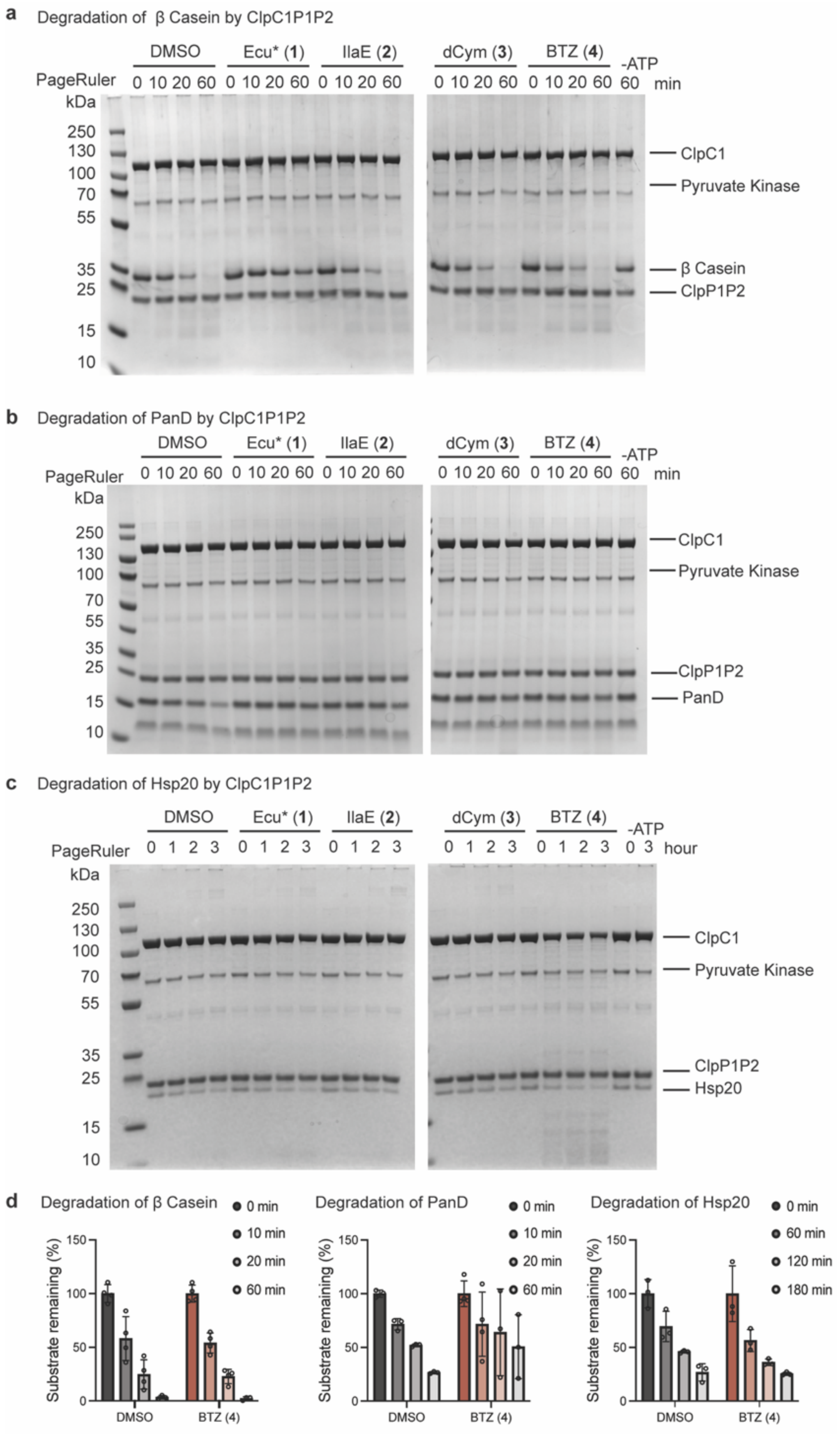
a
Degradation of β Casein by ClpC1P1P2
DMSO
Ecu* (1)
IlaE (2)
dCym (3)
BTZ (4)
-ATP
PageRuler
0 10 20 60 0 10 20 60 0 10 20 60 0 10 20 60 0 10 20 60 60 min
kDa
250
130
100
70
55
35
25
15
10
ClpC1
Pyruvate Kinase
β Casein
ClpP1P2
b
Degradation of PanD by ClpC1P1P2
DMSO
Ecu* (1)
IlaE (2)
dCym (3)
BTZ (4)
-ATP
PageRuler
0 10 20 60 0 10 20 60 0 10 20 60 0 10 20 60 0 10 20 60 60 min
kDa
250
130
100
70
55
35
25
15
10
ClpC1
Pyruvate Kinase
ClpP1P2
PanD
c
Degradation of Hsp20 by ClpC1P1P2
DMSO
Ecu* (1)
IlaE (2)
dCym (3)
BTZ (4)
-ATP
PageRuler
0 1 2 3 0 1 2 3 0 1 2 3 0 1 2 3 0 1 2 3 0 3 hour
kDa
250
130
100
70
55
35
25
15
10
ClpC1
Pyruvate Kinase
ClpP1P2
Hsp20
d
Degradation of β Casein
0 min
10 min
20 min
60 min
Substrate remaining (%)
150
100
50
0
DMSO
BTZ (4)
Degradation of PanD
0 min
10 min
20 min
60 min
Substrate remaining (%)
150
100
50
0
DMSO
BTZ (4)
Degradation of Hsp20
0 min
60 min
120 min
180 min
Substrate remaining (%)
150
100
50
0
DMSO
BTZ (4)

Supplementary Figure. 6| ClpC1P1P2 *in vitro* degradation assays. a-c Exemplar SDS-PAGE gels for ClpC1P1P2 degradation assays with substrates; β-casein (**a**) and PanD (**b**) and Hsp20 (**c**) in the presence of 100 μM drug. Timepoints taken at 0, 10, 20 and 60 minutes for beta-casein and PanD, 0, 60, 120, 180 min for Hsp20. **e** Quantification of *in vitro* degradation assays in presence of BTZ (**4**). Data is presented as mean ± SD, β-Casein n = 4, PanD n = 3, Hsp20 n = 3. Source data and uncropped gels are provided in Source data file.

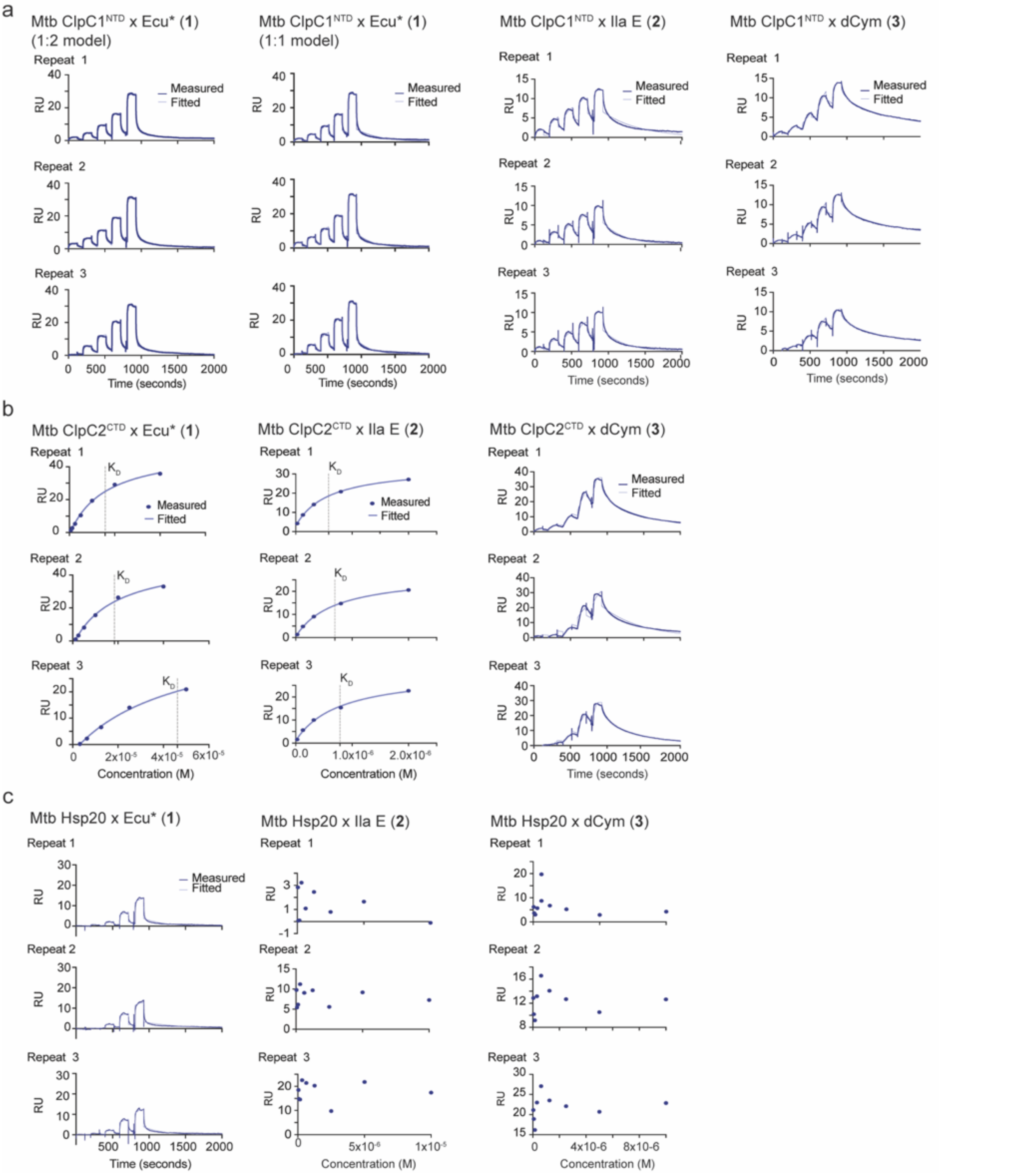

Supplementary Figure. 7| Raw data for surface plasmon resonance (SPR) binding assays shows each NRP could bind another chaperone in addition to ClpC1. a SPR plots for all replicates of NRPs **1-3** ligand binding with recombinant *Mtb* ClpC1-NTD replicates. **b** SPR plots for all replicates of NRPs **1-3** interaction with recombinant *Mtb* ClpC2-CTD. **c** SPR plots for NRP ligand binding to recombinant *Mtb* Hsp20.

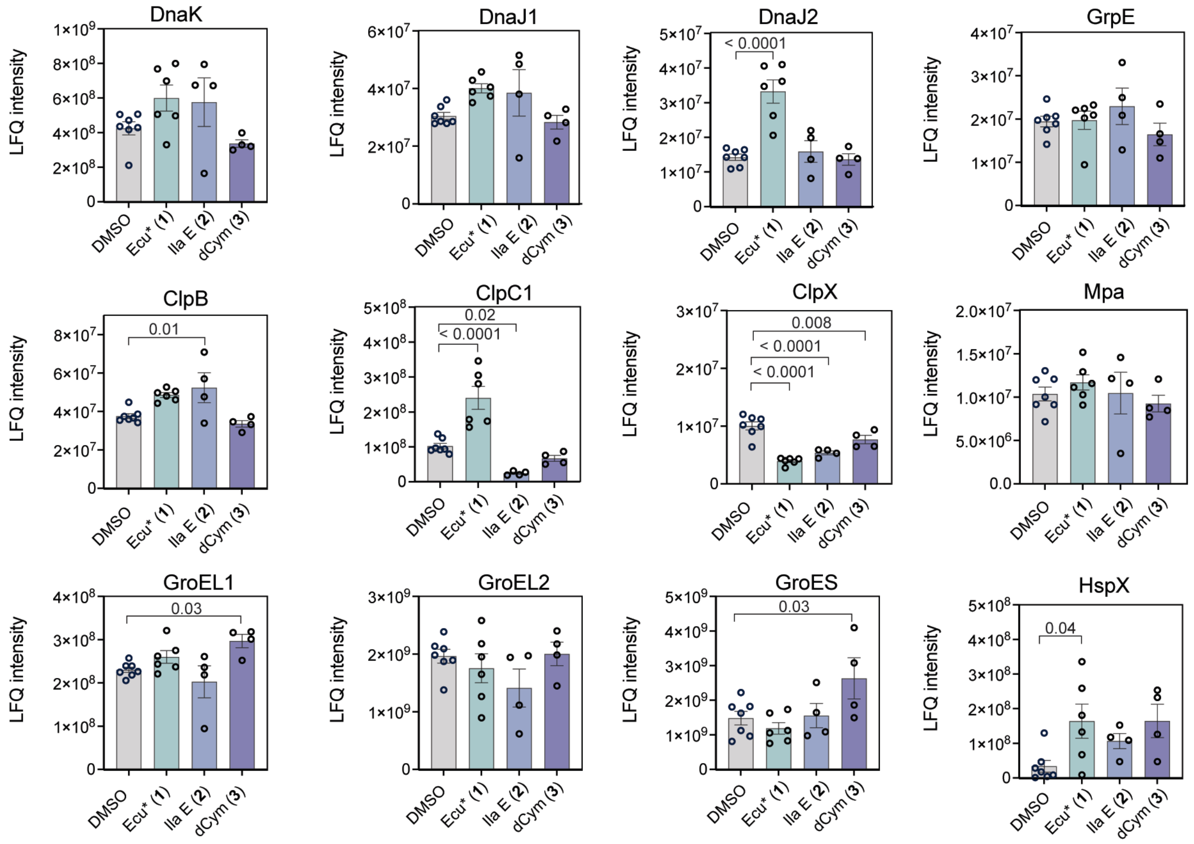

Supplementary Figure. 8 | Key proteins involved in managing stress-induced protein unfolding. LFQ intensities for chaperones following NRP treatment (related to schematic in Fig. 5a). Data is presented as mean ± SEM, DMSO n = 7, **1** n = 6, **2** and **3** n = 4. Data was analysed with BH-adjusted one-way ANOVA. Source data are provided in Supplementary Data 1.

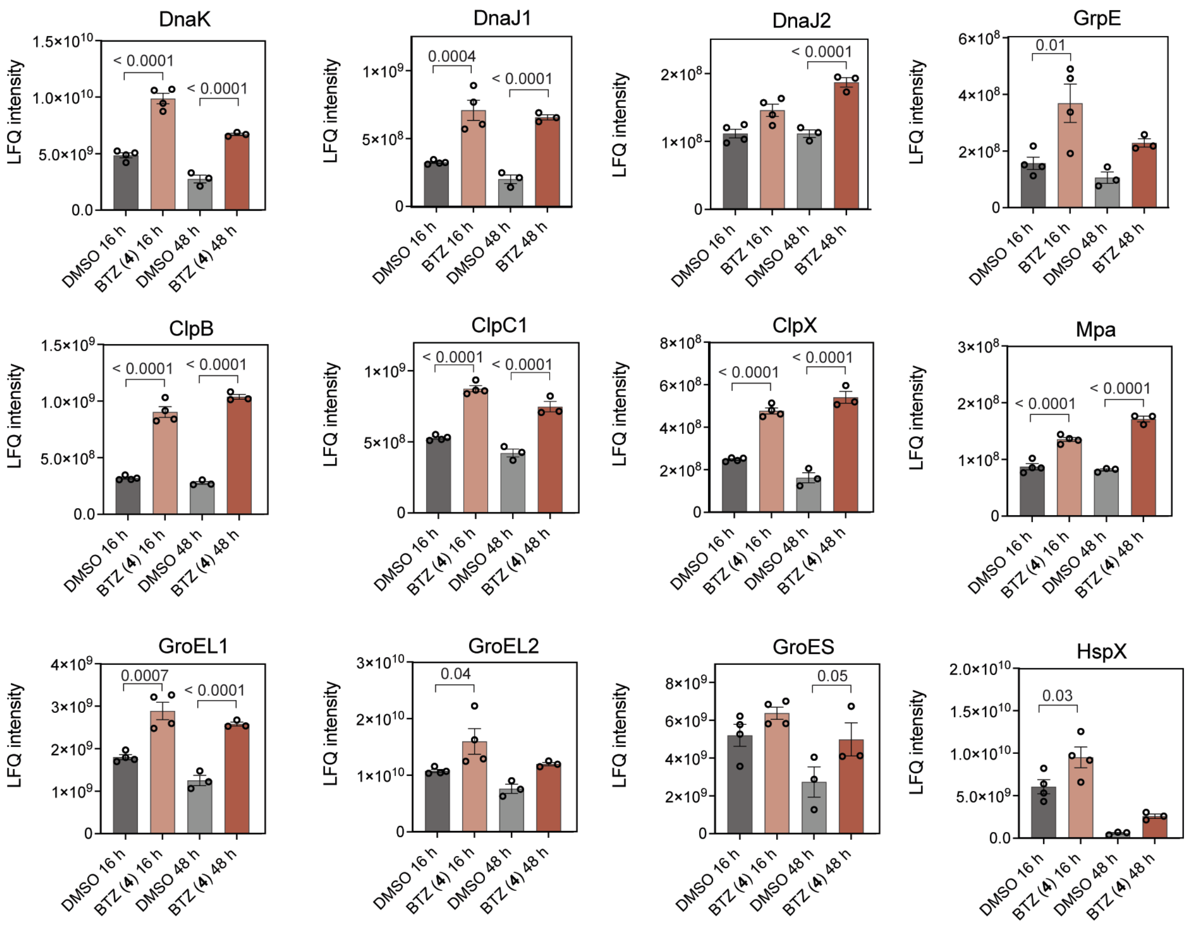

Supplementary Figure. 9| Key proteins involved in managing stress-induced protein unfolding. LFQ intensities for chaperones following BTZ treatment (related to schematic in Fig. 5a). Data is presented as mean ± SEM, 16 h n = 4, 48 h n = 3. Data was analysed with two-way ANOVA and BH adjusted. Source data can be found in Supplementary Data 4.

Supplementary Tables

Supplementary Table 1 | Pearson's χ^2 approximation and two-sided Fisher's exact tests for association between intrinsic disorder and differential abundance following NRP treatment.

	Contingency (downregulated, upregulated)		Pearson's chi-squared test			Fisher's Exact test			
	Disordered (>0.5 disorder score)	Not disordered (<0.05 disorder score)	χ^2	d. f	p	Odds ratio	p	95% confidence interval	Odds ratio does not equal 1
Ecu* (**1**)	34, 3	97, 49	9.402	1	0.002	5.684	0.002	1.66, 30.36	True
Ila E (**2**)	5, 9	87, 32	8.214*	1	0.004	0.207	0.011	0.05, 0.75	True
dCym (**3**)	5, 7	79, 17	10.185*	1	0.001	0.157	0.004	0.03, 0.65	True

Supplementary Table 2 | Pearson's χ^2 approximation and two-sided Fisher's exact tests for association between termini disorder and differential abundance following NRP treatment.

		Contingency (downregulated, upregulated)		Pearson's chi-squared test			Fisher's Exact test			
		Disordered (>70% DPR)	Not disordered (<50% DPR)	χ^2	d. f	p	Odds ratio	p	95% confidence interval	Odds ratio does not equal 1
Ecu* (**1**)	C-term	111, 25	196, 73	3.775	1	0.052	1.652	0.065	0.97, 2.88	False
	N-term	46, 8	305, 105	3.018	1	0.082	1.977	0.093	0.89, 5.01	False
Ila E (**2**)	C-term	64, 33	154, 52	2.518	1	0.113	0.656	0.132	0.38, 1.15	False
	N-term	23, 13	239, 105	0.475	1	0.491	0.778	0.570	0.36, 1.74	False
dCym (**3**)	C-term	45, 21	101, 39	0.341	1	0.559	0.828	0.623	0.42, 1.66	False
	N-term	16, 8	171, 66	0.323	1	0.570	0.773	0.635	0.30, 2.20	False

d. f.: degree of freedom, p.: p-value, * = χ^2 approximation may be incorrect due to small sample size.

If the calculated χ^2 with a d. f. of 1 is > than the critical value for the χ^2 distribution with a d. f of 1 (3.84) then the null hypothesis for independence between the two variables can be rejected.

If the 95% confidence interval for the Fisher's exact test odds ratio does not intercept 1, then the hypothesis that the odds ratio does not equal 1 is considered true.

Supplementary Table 3 | Primers used in this study

name	sequence	description
ClpC2_a_forward	GGGAATGCTCTTGCCGATATCGGA	CRISPRi cloning oligo
ClpC2_a_reverse	AAACTCCGATATCGGCAAGAGCAT	CRISPRi cloning oligo
ClpC2_b_forward	GGGAGTGGCCCAGCCGAAGCGCCTC	CRISPRi cloning oligo
ClpC2_b_reverse	AAACGAGGCGCTTCGGCTGGGCCAC	CRISPRi cloning oligo
MMO759	ACAACTACATCGGGACCGA	qPCR-*Mtb*-ClpC2
MMO760	ATGCGAGCGTGGTGATCA	qPCR-*Mtb*-ClpC2
MMO761	AGCAGCAGGAGATCGACAT	qPCR-*Mtb*-ClpC2
MMO762	AGGTGAGCTCGAGGACCT	qPCR-*Mtb*-ClpC2
ClpC2_CTD_forward	ACTTTAAGAAGGAGATATACC-ATGCAAGGATTCCGCCGCTTTACAC	Protein expression cloning oligo
ClpC2_CTD_reverse	CTTCCTTTCGGGCTTTGTTA-TTAATGGTGATGGTGATGGTGTCCT	Protein expression cloning oligo
Hsp20 _forward	ACTTTAAGAAGGAGATATACC-ATGCATCACCACCATCACCACAATA	Protein expression cloning oligo
Hsp20_reverse	CTTCCTTTCGGGCTTTGTTA-TTACTTTGTAATGGCAATGCGTTGT	Protein expression cloning oligo
PanD_forward	ACTTTAAGAAGGAGATATACC-ATGCATCATCATCATCATCTGCGCA	Protein expression cloning oligo
PanD_reverse	CTTCCTTTCGGGCTTTGTTA-TTAGCCCACGCCCAGGCGCGGATCC	Protein expression cloning oligo

Supplementary Table 4 | Abbreviations

AGC	automatic gain control
ADP	Adenosine diphosphate
ATP	adenosine triphosphate
BCA	bicinchoninic acid assay
Bsb	*bacillus substilis*
CAA	chloroacetamide
CFU	colony forming units
Clp	caseinolytic
CRISPRi	clustered regularly interspaced short palindromic repeats interference
CTD	C-terminal domain
D1	ATPase domain 1
D2	ATPase domain 2
dCym	desoxycyclomarin
DMSO	dimethyl sulfoxide
DPR	disorder promoting residues
DSSP	database of secondary structure assignments
Ecu*	ecumicin analogue
GFP	green florescent protein
HCD	higher energy collision dissociation
Ila E	Ilamycin E2
IPTG	isopropyl β-thiogalactopyranoside
LC-MS/MS	Liquid chromatography with tandem mass spectrometry
LFQ	label free quantification
MDR-TB	multi-drug resistant tuberculosis
MIC_{90}	minimum inhibitory concentration to inhibit 90% bacterial growth
Mtb	*Mycobacterium tuberculosis*
NaCl	sodium chloride
NaPi	sodium phosphate
Ni-NTA	nickel-nitrilotriacetic acid
NRP	natural product antibiotic
NTD	N-terminal domain
pArg	phosphorylated arginine
pLDDTs	predicted local distance difference test
PQC	protein quality control
SDB-RPS	styrenedivinylbenzene-reverse phase sulfonate
SDS	sodium dodecyl sulfate
SDS -PAGE	sodium dodecyl sulfate-polyacrylamide gel electrophoresis
SEC	size exclusion chromatography
SPR	surface plasmon resonance
TB	tuberculosis
TCEP	tris(2-carboxyethyl)phosphine
TFA	trifluoracetic acid
XDR-TB	extremely-drug resistant tuberculosis

Supplementary Information

Chemical synthesis of ilamycin E [IIa E (2)]

General Methods and Materials

All reactions were conducted under an inert atmosphere of argon or nitrogen and at room temperature (25 °C) unless the reaction was performed under aqueous conditions or otherwise stated. Low-temperature baths employed were ice/water (0 °C) and dry ice/acetone (-78 °C). Heating mantels were used for high temperature reactions. Anhydrous THF, MeOH, CH_2Cl_2, DMF, and MeCN were obtained using a PureSolv® solvent purification system. Other anhydrous solvents were purchased from Sigma-Aldrich. Reactions were monitored by thin layer chromatography (TLC) on alumina-backed silica plates (Merck Silica Gel 60 F254). TLC plates were visualised using UV light at λ = 254 nm and staining with solutions of ninhydrin, vanillin, potassium permanganate, or phosphomolybdic acid (PMA) followed by heating. Silica flash chromatography (Velocity Scientific ultra-pure irregular silica, 40-63 µm, 60 Å) was employed to purify crude reaction mixtures using eluent systems as specified.

All commercially available reagents were used as obtained from Sigma-Aldrich, Merck, AK Scientific, or Combi-Blocks. Amino acids, coupling reagents, and 2-CTC resin were obtained from NovaBiochem or Mimotopes. Peptide synthesis grade DMF was obtained from Ajax, Merck, or Labscan. Fmoc-SPPS was conducted in fritted syringes purchased from Torviq. HPLC grade MeCN was obtained from Sigma Aldrich. All non-commercially available reagents were synthesised according to literature procedure as referenced.

NMR spectra were obtained at 300 K (unless otherwise stated) using Bruker Avance DRX300, DPX400, DPX500, or DPX600 spectrometers at frequencies of 300 MHz, 400 MHz, 500 MHz, or 600 MHz respectively in $CDCl_3$, CD_3OD, or $(CD_3)_2SO$ unless otherwise specified. Coupling constants are reported in Hertz (Hz). ^1H NMR chemical shifts are reported in parts per million (ppm) and are referenced to solvent residual signals for $CDCl_3$ (δ = 7.26 ppm), CD_3OD (δ = 3.31 ppm), or $(CD_3)_2SO$ (δ = 2.50 ppm). ^1H NMR data is reported as follows: chemical shift values (ppm), multiplicity (s = singlet, d = doublet, t = triplet, q = quartet, m = multiplet), coupling constant(s) and relative integral. ^{13}C NMR spectra were obtained using Bruker Avance DRX300, DPX400, DPX500, or DPX600 spectrometers at frequencies of 75 MHz, 100 MHz, 125 MHz, or 150 MHz in $CDCl_3$, CD_3OD, or $(CD_3)_2SO$ unless otherwise specified. ^{13}C NMR data is reported as chemical shift values (ppm).

Infrared (IR) absorption spectra were recorded on a Bruker Alpha Spectrometer with attenuated total reflection (ATR) capability and were processed on OPUS 4 software. Optical rotations were measured at 23 °C at 589 nm (Na D line) using a Perkin Elmer Model 341 polarimeter, with concentrations (c) reported in g/100 mL. Low resolution mass spectra (LRMS) were recorded using a Bruker amaZon SL mass spectrometer or Shimadzu 2020 (ESI) mass spectrometer. High resolution mass spectra (HRMS)

were recorded using a Bruker Apex Qe 7T Fourier Transform Ion Cyclotron Resonance Mass Spectrometer (FTICR) mass spectrometer.

UPLC-MS was performed either on a Shimadzu UPLC-MS instrument with an LC-M20A pump, SPD-M30A diode array detector, or using a Shimadzu 2020 (ESI) mass spectrometer operating in positive mode. Separations on the UPLC system were performed on a Waters Acquity 1.7 μm, 2.1 × 50 mm (C18) column using a mobile phase of 0.1 vol% formic acid in water (solvent A) and 0.1 vol% formic acid in MeCN (solvent B) using linear gradients. Preparative reversed-phase HPLC was performed using a Waters 2535 Multisolvent Delivery System with a Waters 2489 UV/visible detector operating at 214 nm and 280 nm.

Synthetic details and analytical data

*Synthesis of methyl (tert-butoxycarbonyl)-L-tryptophanate (**S1**)*

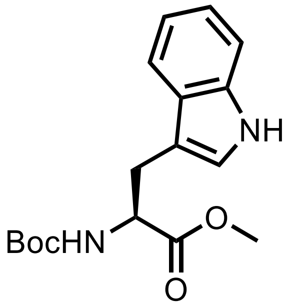

To a solution of L-Trp-OMe·HCl (7.64 g, 30.0 mmol) in CH_2Cl_2 (100 mL) was added Et_3N (8.4 mL, 60 mmol) and Boc_2O (8.4 mL, 37 mmol) and was stirred at rt for 4 h. The mixture was concentrated *in vacuo*, diluted with H_2O (100 mL) and extracted with EtOAc (3 × 100 mL). The combined organic layers were then washed with brine, dried with Na_2SO_4, and concentrated *in vacuo*. The crude residue was purified by flash chromatography (1→50 vol% EtOAc in petroleum benzine) to afford title compound **S1** as a white solid (8.59 g, 90%).

^{1}H NMR (300 MHz, $CDCl_3$) δ 8.39 (s, 1H), 7.58 (dd, *J* = 7.5, 1.3 Hz, 1H), 7.36 (d, *J* = 8.0 Hz, 1H), 7.21 (td, *J* = 7.5, 1.3 Hz, 1H), 7.14 (td, *J* = 7.4, 1.2 Hz, 1H), 5.15 (d, *J* = 8.2 Hz, 1H), 4.68 (q, *J* = 6.3 Hz, 1H), 3.70 (s, 3H), 3.32 (d, *J* = 5.5 Hz, 2H), 1.46 (s, 9H). **^{13}C NMR** (75 MHz, $CDCl_3$) δ 172.9, 155.4, 136.3, 127.7, 122.9, 122.2, 119.6, 118.8, 111.4, 110.1, 80.0, 54.3, 52.3, 28.4, 28.1. **LRMS** (ESI+): Mass calculated for $C_{17}H_{22}N_2O_4$: 319.2 $[M+H]^+$. Mass found: *m/z* = 319.2 $[M+H]^+$. **HRMS** (ESI+): Mass calculated for $C_{17}H_{22}N_2O_4$: 319.1652 $[M+H]^+$. Mass found: *m/z* = 319.1647 $[M+H]^+$. **IR** ν_{max} 3345, 2977, 1602, 1501 cm^{-1}. **$[\alpha]_D$** = -1.80 ° (*c* 1.0, $CHCl_3$). These data are in agreement with those reported by Speckmeier *et al.* [7]

*Synthesis of methyl Nα-(tert-butoxycarbonyl)-1-(2-methylbut-3-en-2-yl)-L-tryptophanate (**S2**)*

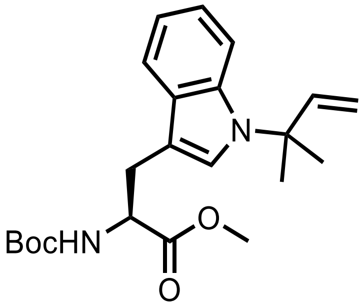

Boc-L-Trp-OMe (**S1**, 6.30 g, 19.8 mmol), $Cu(OAc)_2$ (7.36 g, 40.5 mmol), AgTFA (8.95 g, 40.5 mmol), and $Pd(OAc)_2$ (0.89 g, 4.0 mmol) were dissolved in anhydrous, anoxic MeCN (150 mL). 2-methyl-2-butene (46 mL, 435 mmol) was then added to the solution and was heated to 35 °C for 4 h with addition of a further two portions of $Pd(OAc)_2$ (2 × 0.89 g, 4.0 mmol). The solution was concentrated under reduced pressure, loaded onto celite, and dried under vacuum overnight. The crude residue was passed through a Buchner funnel containing a layer of filter paper, sand, silica, sand, and filter paper using 0-50 vol% EtOAc in petroleum benzine as eluent. The filtrate was then concentrated under reduced pressure. The crude product was partitioned between Et_2O (400 mL) and EDTA (50 mM in H_2O, 500 mL) and the aqueous layer was extracted with Et_2O (2 × 400 mL) the combined organic extractions were concentrated under reduced pressure. The crude residue was purified by column chromatography (0→60 vol% EtOAc in petroleum benzine) to afford title compound **S2** as a yellow oil (3.97 g, 52%).

^{1}H NMR (300 MHz, $CDCl_3$) δ 7.57 – 7.46 (m, 2H), 7.15 – 7.05 (m, 3H), 6.14 (dd, *J* = 17.5, 10.7 Hz, 1H), 5.30 – 5.11 (m, 2H), 4.66 (q, *J* = 6.5 Hz, 1H), 3.68 (s, 3H), 3.36 – 3.16 (m, 2H), 1.73 (s, 6H), 1.44 (s, 9H). **^{13}C NMR** (75 MHz, $CDCl_3$) δ 173.0, 155.3, 144.2, 135.6, 129.8, 123.9, 121.0, 119.1, 118.9, 113.9, 113.6, 108.2, 79.8, 59.1, 54.5, 52.2, 28.5, 28.3, 28.1, 28.0. **LRMS** (ESI+): Mass calculated for $C_{22}H_{30}N_2O_4$: 387.2 $[M+H]^+$. Mass found: *m/z* = 387.2 $[M+H]^+$. **HRMS** (ESI+): Mass calculated for $C_{22}H_{30}N_2O_4$: 409.2098 $[M+Na]^+$. Mass found: 409.2094 $[M+Na]^+$. **IR** ν_{max} 3431, 2980, 1709, 1500 cm^{-1}. **$[\alpha]_D$** = +2.4° (*c* 1.1, $CHCl_3$). These data are in agreement with those reported by Luzung, *et al.* [8]

*Synthesis of methyl N^{α}-(((9H-fluoren-9-yl)methoxy)carbonyl)-1-(2-methylbut-3-en-2-yl)-L-tryptophanate (**S3**)*

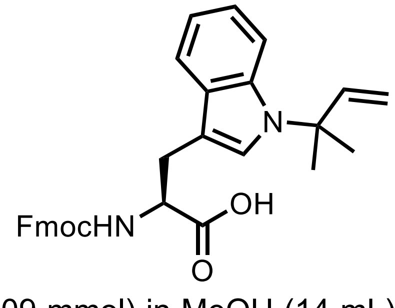

A solution of **S2** (807 mg, 2.09 mmol) in MeOH (14 mL) was cooled to 0 ºC followed by the dropwise addition of thionyl chloride (0.23 mL, 3.1 mmol), after which the reaction was heated to 50 °C and stirred for 2 h. The mixture was then diluted with CH_2Cl_2 (30 mL) and H_2O (30 mL) and basified by dropwise addition of aqueous 1 M

NaOH (5 mL). The organic layer was separated, washed with water (20 mL) and brine (30 mL), dried over Na_2SO_4, and concentrated *in vacuo*. The crude residue was then dissolved in THF (100 mL) and cooled to 0 °C before the dropwise addition of an aqueous solution of LiOH (26 mL, 1.2 M) and the reaction was stirred at this temperature for 1.5 h. The biphasic mixture was neutralised with aqueous 1 M HCl (30 mL) and diluted with saturated aqueous $NaHCO_3$ (70 mL), followed by the addition of Fmoc *N*-hydroxysuccinimide ester (594 mg, 2.3 mmol). The mixture was warmed to rt and stirred for 12 h, after which the crude reaction mixture was diluted with EtOAc (80 mL) and acidified to pH <5 using H_2SO_4. The organic layer was separated, washed with water (50 mL) and brine (50 mL), dried over Na_2SO_4 and concentrated *in vacuo*. The crude residue was purified by flash column chromatography (0→10 vol% MeOH in CH_2Cl_2) to afford title compound **S3** as a pale-yellow foam (880 mg, 85% over 3 steps).

^{1}H NMR (400 MHz, CDCl3) δ 7.75 (d, *J* = 7.5 Hz, 2H), 7.64 – 7.46 (m, 4H), 7.43 – 7.35 (m, 2H), 7.31 – 7.25 (m, 3H), 7.16 (s, 1H), 7.15 – 7.06 (m, 2H), 6.11 (dd, *J* = 17.5, 10.6 Hz, 1H), 5.34 (d, *J* = 8.2 Hz, 1H), 5.20 (d, *J* = 10.7 Hz, 1H), 5.13 (d, *J* = 17.4 Hz, 1H), 4.82 – 4.73 (m, 1H), 4.42 – 4.31 (m, 2H), 4.19 (dd, *J* = 7.3, 7.3 Hz, 1H), 3.43 – 3.28 (m, 2H), 1.71 (s, 6H). **LRMS** (+ESI) Mass calculated for $C_{31}H_{30}N_2O_4$: 495.2 $[M+H]^+$. Mass found: *m/z* = 495.0 $[M+H]^+$. **$[\alpha]_D$** = +21.4° (*c* 0.8, $CHCl_3$). These data are in agreement with those previously reported by Baran *et al.* [9]

*Synthesis of 1-(tert-butyl) 4-(4,5,6,7-tetrachloro-1,3-dioxoisoindolin-2-yl) (tert-butoxycarbonyl)-L-aspartate (**S4**)*

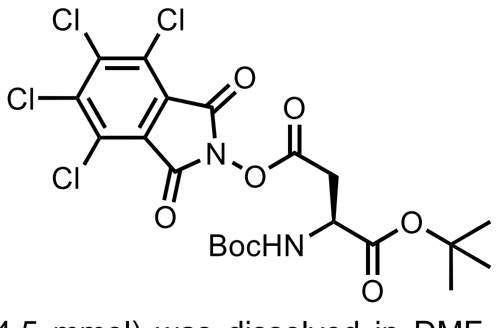

Boc-Asp-OtBu (1.32 g, 4.5 mmol) was dissolved in DMF (50 mL) followed by the addition of CITU (2.72 g, 5.3 mmol) and NMM (1.0 mL, 10.0 mmol). The reaction mixture was stirred at rt for 2 h after which it was diluted with EtOAc (250 mL) and washed with H_2O (4 × 200 mL), 0.5 M HCl (100 mL), and brine (100 mL). The organic layer was then dried over $NaSO_4$, filtered, and concentrated *in vacuo.* The resulting crude residue was purified by flash chromatography (1→20 vol% EtOAc in petroleum benzine) to afford title compound **S4** as an off-white foam (2.29 g, 88%).

^{1}H NMR (300 MHz, $CDCl_3$) δ 5.47 (d, *J* = 7.7 Hz, 1H), 4.56 (dt, *J* = 8.3, 4.6 Hz, 1H), 3.32 (dd, *J* = 17.3, 4.5 Hz, 1H), 3.23 (dd, *J* = 17.3, 4.8 Hz, 1H), 1.47 (s, 9H), 1.46 (s, 9H). **^{13}C NMR** (75 MHz, $CDCl_3$) δ 168.6, 167.1, 157.2, 155.3, 141.1, 130.5, 124.6, 83.5, 80.4, 50.2, 34.1, 28.3, 27.8. **LRMS** (ESI+): Mass calculated for $C_{21}H_{22}Cl_4N_2O_8$:

593.0 [M+Na]$^+$. Mass found: *m/z* = 595.00 [M+Na]$^+$. **HRMS** (ESI+): Mass calculated for $C_{21}H_{22}Cl_4N_2O_8$: 593.0023 [M+Na]$^+$. Mass found: *m/z* = 593.0018 [M+Na]$^+$. **IR** ν_{max} 3433, 2979, 2934, 1821, 1795, 1747, 1709 cm^{-1}. **[α]$_D$** = +31.1° (*c* 1.0, $CHCl_3$).

*Synthesis of tert-butyl (S,E)-2-((tert-butoxycarbonyl)amino)hex-4-enoate (**S5**)*

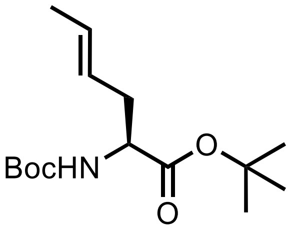

To a round-bottom flask was added Mg (600 mg, 25 mmol), which was stirred in 1 M HCl (20 mL) for 5 min, after which the supernatant was decanted and the remaining solid washed with water (20 mL), acetone (20 mL), Et_2O (20 mL), and dried *in vacuo*. Separately, LiCl (530 mg, 12.5 mmol) was dried under vacuum with a heat gun and added to the M, followed by anhydrous THF (2 mL) and a granule of iodine. A solution of *trans*-1-bromo-1-propene (1.3 mL, 14.9 mmol) in anhydrous THF (12 mL) was then added dropwise to this mixture at rt, after which this reaction mixture was stirred at 80 °C for 1 h. $ZnCl_2$ (950 mg, 7.0 mmol) was dried under vacuum with a heat gun then dissolved in anhydrous THF (7 mL) with vigorous stirring. The solution of freshly prepared alkenyl Grignard reagent (10.9 mL, 0.64 M, 7.0 mmol, concentration was determined by titrating against salicylaldehyde phenylhydrazone) was added dropwise to the $ZnCl_2$ and stirred at rt for 15 min. In a separate flask, redox-active ester **S4** (1.82 g, 3.2 mmol), $Ni(acac)_2{\cdot}xH_2O$ (182 mg, 0.64 mmol, 20 mol.%), $MgBr_2{\cdot}OEt_2$ (1.61 g, 6.4 mmol), and 2,2'-bipyridine (106 mg, 0.64 mmol) were dissolved in anhydrous DMF (30 mL). The organozinc solution (19 mL, 0.34 M, 6.4 mmol) was then added and the resulting reaction mixture was stirred at rt for 16 h. The mixture was diluted with EtOAc (30 mL) and acidified with 1 M HCl (25 mL). The organic layer was collected and the aqueous layer was extracted with EtOAc (3 × 50 mL), after which the organic extractions were combined, washed with brine (50 mL), dried over Na_2SO_4, filtered, and concentrated *in vacuo*. The crude residue was purified by flash chromatography (1→20 vol% EtOAc in petroleum benzine) to afford title compound **S5** as a colourless oil (560 mg, 62%).

^{1}H NMR (500 MHz, $CDCl_3$) δ 5.57 – 5.46 (m, 1H), 5.37 – 5.24 (m, 1H), 5.01 (d, *J* = 8.2 Hz, 1H), 4.21 – 4.14 (m, 1H), 2.47 – 2.33 (m, 2H), 1.65 (dd, *J* = 6.4, 1.5 Hz, 3H), 1.45 (s, 9H), 1.43 (s, 9H). **^{13}C NMR** (126 MHz, $CDCl_3$) δ 171.5, 155.3, 129.6, 125.0, 81.8, 79.7, 53.8, 36.0, 28.5, 28.2, 18.1. **LRMS** (ESI+): Mass calculated for $C_{15}H_{27}NO_4$: 308.2 [M+Na]$^+$. Mass found: *m/z* = 308.1 [M+Na]$^+$. **HRMS** (ESI+): Mass calculated for $C_{15}H_{27}NO_4$: 308.1832 [M+Na]$^+$. Mass found: 308.1829 [M+Na]$^+$. **IR** ν_{max} 3361, 2977, 2932, 1712 cm^{-1}. **[α]$_D$** = +11.2° (*c* 1.0, $CHCl_3$).

*Synthesis of (S,E)-2-((((9H-fluoren-9-yl)methoxy)carbonyl)amino)hex-4-enoic acid (**S6**)*

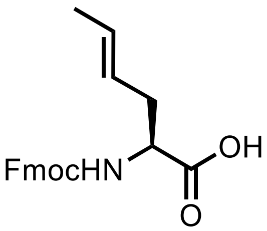

S5 (350 mg, 1.22 mmol) was dissolved in H_2O/TFA (1:19 v/v, 8 mL) and stirred at rt for 5 h. The solution was concentrated *in vacuo* and the crude residue diluted with sat. aqueous $NaHCO_3$ (21 mL) to pH ~9. THF (21 mL) and Fmoc-OSu (500 mg, 1.47 mmol) were added to the solution and stirred at rt overnight. The solution was acidified to pH ~1 with 1 M HCl and extracted with EtOAc (4 × 50 mL). The combined organic layers were washed with brine, dried with Na_2SO_4, filtered, and concentrated *in vacuo*. The crude product was purified by flash chromatography (1→10 vol% MeOH in CH_2Cl_2) to afford title compound **S6** as an off-white foam (370 mg, 85%).

^{1}H NMR (500 MHz, MeOD) δ 7.78 (d, *J* = 7.6 Hz, 2H), 7.69 – 7.58 (m, 2H), 7.38 (t, *J* = 7.5 Hz, 2H), 7.30 (td, *J* = 7.5, 1.2 Hz, 2H), 5.64 – 5.52 (m, 1H), 5.47 – 5.36 (m, 1H), 4.37 – 4.29 (m, 2H), 4.21 (t, *J* = 7.0 Hz, 1H), 4.17 (dd, *J* = 8.4, 5.0 Hz, 1H), 2.56 – 2.44 (m, 1H), 2.44 – 2.31 (m, 1H), 1.65 (dd, *J* = 6.4, 1.5 Hz, 3H). **^{13}C NMR** (126 MHz, MeOD) δ 175.4, 158.5, 145.3, 145.2, 142.6, 129.9, 128.8, 128.1, 128.1, 127.0, 126.3, 120.9, 68.0, 55.5, 35.9, 18.1. **LRMS** (ESI+): Mass calculated for $C_{21}H_{21}NO_4$: 352.4 $[M+H]^+$. Mass found: *m/z* = 352.0 $[M+H]^+$. **HRMS** (ESI+): Mass calculated for $C_{21}H_{21}NO_4$: 374.1363 $[M+Na]^+$. Mass found: *m/z* = 374.1357 $[M+Na]^+$. **IR** ν_{max} 3307, 3021, 2942, 1705, 1514 cm^{-1}. $[\alpha]_D$ = +115.0° (*c* 1.0, $CHCl_3$).

*Synthesis of 1-allyl 5-(tert-butyl) (tert-butoxycarbonyl)-L-glutamate (**S7**)*

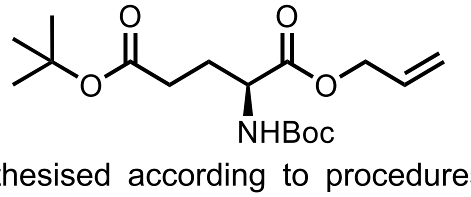

This compound was synthesised according to procedures previously reported by Cergol *et al.* [10]

*Synthesis of 1-allyl 5-(tert-butyl) (2S,4S)-2-((tert-butoxycarbonyl)amino)-4-methylpentanedioate (**S8**)*

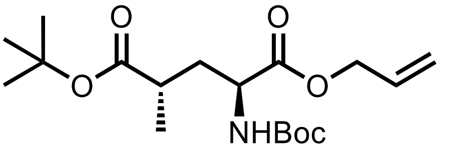

A solution of hexamethyldisilazane (3.6 ml, 17 mmol) in deoxygenated THF (40 mL) was cooled to 0 °C and *n*-butyl lithium (6.9 mL, 17 mmol, 2.5 M in THF) was added dropwise. The reaction mixture was stirred at this temperature for 30 min before cooling to -78 °C. A solution of Boc-Glu(O*t*Bu)-OAll (**S7**) (2.37 g, 6.90 mmol) in

deoxygenated THF was cooled to -78 °C and added dropwise to the LiHMDS solution by transfer cannula. The reaction mixture was stirred at -78 °C for 30 min before the dropwise addition of methyl iodide (570 µL, 9.0 mmol). The resultant reaction was stirred at this temperature for 4.5 h and quenched by the dropwise addition of MeOH (20 mL). Following warming to rt, the solution was diluted with 1 M HCl (50 ml) and the aqueous layer extracted with EtOAc (3 × 60 ml). The combined organic layers were washed with water (30 ml) and brine (30 mL), dried over anhydrous $MgSO_4$, filtered, and concentrated *in vacuo.* The crude residue was purified by flash column chromatography (2→15 vol% EtOAc in petroleum benzine) to afford a single diastereomer of title compound **S8** as a pale-yellow oil. (2.17 g, 88%).

^{1}H NMR (400 MHz, $CDCl_3$) δ 5.86 (ddt, *J* = 17.3, 10.5, 5.7 Hz, 1H), 5.28 (dq, *J* = 17.1, 1.5 Hz, 1H), 5.20 (dq, *J* = 10.4, 1.3 Hz, 1H), 4.97 (d, *J* = 9.0 Hz, 1H), 4.63 – 4.52 (m, 2H), 4.32 – 4.20 (m, 1H), 2.45 – 2.32 (m, 1H), 1.96 (ddd, *J* = 14.1, 10.3, 7.5 Hz, 1H), 1.77 (dt, *J* = 14.1, 5.7 Hz, 1H), 1.40 (s, 9H), 1.38 (s, 9H), 1.13 (d, *J* = 7.0 Hz, 3H). **^{13}C NMR** (101 MHz, $CDCl_3$) δ 175.6, 172.5, 155.6, 131.7, 118.7, 80.6, 79.9, 65.9, 52.5, 37.8, 35.4, 28.3, 28.1, 17.6. **LRMS** (+ESI): Mass calculated for $C_{18}H_{31}NO_6$: 380.2 $[M+H]^+$. Mass found: *m/z* = 380.1 $[M+Na]^+$. **HRMS** (+ESI): Mass calculated for $C_{18}H_{31}NO_6$: 380.2049 $[M+Na]^+$. Mass found: 380.2043 $[M+Na]^+$. **IR** ν_{max} (ATR) 2977, 2936, 1724, 1367, 1253, 1153 cm^{-1}. **$[\alpha]_D$** = -95 ° (*c* 1.90, $CHCl_3$).

*Synthesis of 1-allyl 5-(tert-butyl) (2S,4S)-2-((tert-butoxycarbonyl)(methyl)amino)-4-methylpentanedioate (**S9**)*

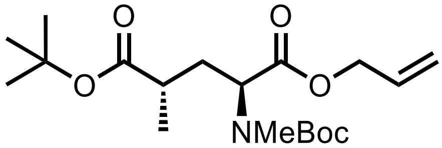

A solution of **S8** (1.78 g, 4.9 mmol) and methyl iodide (1.4 mL, 20 mmol) in DMF (40 mL) was added dropwise to a suspension of sodium hydride (60% in mineral oil, 240 mg, 6 mmol) in DMF (15 mL) at 0 °C. The mixture was slowly warmed to room temperature and stirred for 75 min, after which the reaction was quenched by the addition of sat. aqueous NH_4Cl (6 mL) and water (10 mL) and the aqueous layer was extracted into Et_2O (3 × 80 mL). The combined organic layers were washed with water to remove DMF (5 × 150 mL) and brine (50 mL), dried over Na_2SO_4, filtered, and concentrated *in vacuo*. The crude residue was purified by flash chromatography (2→15 vol% EtOAc in petroleum benzine) to afford title compound **S9** as a pale-yellow oil (1.65 g, 91%).

^{1}H NMR (400 MHz, $CDCl_3$, mixture of rotamers) δ 5.86 – 5.74 (m, 1H), 5.21 (d, *J* = 17.2 Hz, 1H), 5.13 (t, *J* = 8.8 Hz, 1H), 4.72 (dd, *J* = 11.1, 5.0 Hz, 0.5H), 4.51 (d, *J* = 5.6 Hz, 2H), 4.38 (dd, *J* = 10.6, 5.2 Hz, 0.5H), 2.73 (s, 1.5H), 2.68 (s, 1.5H), 2.27 – 2.15 (m, 1H), 2.13 – 1.98 (m, 1H), 1.91 – 1.77 (m, 1H), 1.37 – 1.32 (m, 18H), 1.06 (d, *J* = 7.0 Hz, 3H). **^{13}C NMR** (126 MHz, $CDCl_3$, mixture of rotamers) δ 175.4, 175.2, 171.4, 171.1, 156.3, 155.4, 131.9, 131.7, 118.5, 118.3, 80.5, 80.4, 80.3, 80.2, 65.7,

65.6, 57.7, 56.3, 37.5, 37.4, 32.9, 32.2, 31.3, 30.8, 28.4, 28.1, 16.6. **LRMS** (+ESI): Mass calculated for $C_{19}H_{33}NO_6$: 394.2 $[M+Na]^+$. Mass found: *m/z* = 394.2 $[M+Na]^+$. **HRMS** (+ESI): Mass calculated for $C_{19}H_{33}NO_6$: 394.2206 $[M+Na]^+$. Mass found: 394.2199 $[M+Na]^+$. **IR** ν_{max} (ATR) 2977, 1730, 1699, 1367, 1152 cm^{-1}. **[α]D** = -6.7° (*c* 1.35, $CHCl_3$).

*Synthesis of (2S,4S)-4-((((9H-fluoren-9-yl)methoxy)carbonyl)(methyl)amino)-5-(allyloxy)-2-methyl-5-oxopentanoic acid (**S10**)*

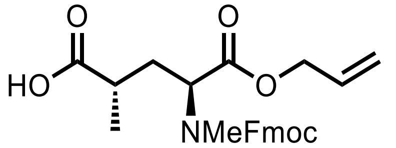

Compound **S9** (3.34 g, 9.0 mmol) was dissolved in a solution of trifluoroacetic acid (38 mL) and water (2 mL) and stirred at room temperature for 2 h, after which volatiles were removed under a stream of nitrogen. The crude residue was dissolved in THF (45 mL) and sat. aqueous $NaHCO_3$ (45 mL) followed by the addition of Fmoc *N*-hydroxysuccinimide ester (3.63 g, 10.8 mmol) and the reaction mixture was stirred at room temperature for 16 h. The reaction was quenched by acidification with 1 M HCl (30 mL) and extracted with EtOAc (3 × 60 mL). The combined organic layers were washed with 1 M HCl (40 mL), water (40 mL), and brine (40 mL) before they were dried over $MgSO_4$, filtered, and concentrated *in vacuo*. The crude residue purified by flash chromatography (10→50 vol% EtOAc in petroleum benzine with 1 vol% AcOH) to afford title compound **S10** as a colourless oil (3.62 mg, 92% over 2 steps).

^{1}H NMR (400 MHz, $CDCl_3$, mixture of rotamers) δ 7.76 (t, *J* = 7.8 Hz, 2H), 7.60 (d, *J* = 7.4 Hz, 1.2H), 7.54 (t, *J* = 6.6 Hz, 0.8H), 7.45 – 7.27 (m, 4H), 5.97 – 5.78 (m, 1H), 5.34 – 5.26 (m, 1H), 5.26 – 5.21 (m, 1H), 4.94 (dd, *J* = 11.3, 4.7 Hz, 1H), 4.67 – 4.56 (m, 2H), 4.52 – 4.38 (m, 3H), 4.27 (t, *J* = 6.9 Hz, 0.6H), 4.22 (t, *J* = 5.7 Hz, 0.4H), 2.84 (s, 1.8H), 2.80 (s, 1.2H), 2.43 – 2.00 (m, 2.6H), 1.89 – 1.79 (m, 0.4H), 1.24 (d, *J* = 7.0 Hz, 1.8H), 0.99 (d, *J* = 6.9 Hz, 1.2H). **^{13}C NMR** (101 MHz, $CDCl_3$, mixture of rotamers) δ 181.5, 181.1, 170.8, 170.3, 157.2, 156.4, 144.0, 143.9, 141.5, 131.7, 131.6, 127.9, 127.8, 127.8, 127.7, 127.2, 125.1, 124.8, 120.1, 120.1, 118.9, 118.9, 68.0, 67.7, 66.1, 66.0, 57.0, 56.8, 47.4, 47.3, 36.4, 36.3, 32.3, 32.0, 31.2, 30.6, 16.3, 16.3. **LRMS** (+ESI): Mass calculated for $C_{25}H_{27}NO_6$: 460.2 $[M+Na]^+$. Mass found: *m/z* = 460.0 $[M+Na]^+$. **HRMS** (+ESI): Mass calculated for $C_{25}H_{27}NO_6$: 460.1736 $[M+Na]^+$. Mass found: 460.1743 $[M+Na]^+$. **IR** ν_{max} (ATR) 2975, 1739, 1703, 1451, 1315, 1258, 1197, 1156 cm^{-1}. **[α]D** = -6.4° (*c* 1.19, $CHCl_3$).

*Synthesis of allyl (2S,4S)-2-((((9H-fluoren-9-yl)methoxy)carbonyl)(methyl)amino)-5-hydroxy-4-methylpentanoate (**S11**)*

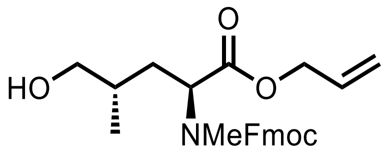

Carboxylic acid **S10** (2.62 g, 6.0 mmol) was dissolved in THF (60 mL) and cooled to 0 °C. *iso*-Butyl chloroformate (2.3 mL, 18.0 mmol) was added dropwise followed by *i*-Pr_2NEt (1.7 mL, 9.0 mmol) and the reaction mixture was warmed to room temperature and stirred for 1 h. The reaction was then cooled to 0 °C and sodium borohydride (1.67 g, 42.0 mmol) was added, followed by the dropwise addition of water (20 mL). The reaction mixture was stirred at this temperature for a further 1 h, after which it was warmed to room temperature and the organic solvent was removed under a stream of nitrogen. The remaining aqueous suspension was extracted with EtOAc (3 × 80 mL). The combined organic layers were washed with saturated aqueous $NaHCO_3$ (50 mL), 1 M HCl (40 mL), water (40 mL), and brine (40 mL) before they were dried over Na_2SO_4, filtered, and concentrated *in vacuo*. The crude residue was purified by flash chromatography (20→60 vol% EtOAc in petroleum benzine) to afford title compound **S11** as a colourless oil (2.44 g, 96%).

^{1}H NMR (200 MHz, $CDCl_3$, mixture of rotamers) δ 7.61 (d, *J* = 7.4 Hz, 2H), 7.44 (t, *J* = 8.7 Hz, 2H), 7.32 – 7.08 (m, 4H), 5.91 – 5.56 (m, 1H), 5.26 – 5.00 (m, 2H), 4.89 (dd, *J* = 11.5, 4.6 Hz, 1H), 4.56 – 4.00 (m, 5H), 3.61 – 2.90 (m, 3H), 2.74 (s, 1.8H), 2.72 (s, 1.2H), 2.01 – 0.96 (m, 3H), 0.90 – 0.75 (m, 1.8H), 0.69 – 0.53 (m, 1.2H). **^{13}C NMR** (50 MHz, $CDCl_3$, mixture of rotamers) δ 171.5, 171.2, 157.0, 156.4, 143.9, 143.9, 143.8, 141.2, 131.6, 127.6, 127.0, 124.9, 124.6, 119.9, 118.4, 67.7, 67.6, 65.6, 56.1, 47.1, 32.4, 32.2, 32.1, 31.9, 15.6, 15.2. **LRMS** (+ESI): Mass calculated for $C_{25}H_{29}NO_5$: 426.2 $[M+Na]^+$. Mass found: *m/z* = 424.5 $[M+Na]^+$. **HRMS** (+ESI): Mass calculated for $C_{25}H_{29}NO_5$: 446.1944 $[M+Na]^+$. Mass found: 446.1950 $[M+Na]^+$. **IR** ν_{max} (ATR) 3485, 2955, 2874, 1740, 1699, 1478, 1329, 1198, 1158 cm^{-1}. **$[\alpha]_D$** = -20.9° (*c* 0.93, $CHCl_3$).

*Synthesis of allyl (2S,4S)-2-((((9H-fluoren-9-yl)methoxy)carbonyl)(methyl)amino)-5-((tert-butyldiphenylsilyl)oxy)-4-methylpentanoate (**S12**)*

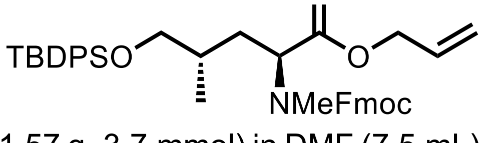

A solution of alcohol **S11** (1.57 g, 3.7 mmol) in DMF (7.5 mL) was cooled to 0 ºC before the addition imidazole (1.25 g, 18.4 mmol) and dropwise addition of *tert*-butyldiphenylsilyl chloride (1.6 mL, 6.1 mmol). The reaction mixture was stirred at room temperature for 16 h, after which the solvent was removed *in vacuo* and the crude residue suspended in 1 M HCl (40 mL) and extracted into diethyl ether (3 × 60 mL). The combined organic layers were washed with brine (40 mL), dried over Na_2SO_4, filtered and concentrated *in vacuo*. The crude residue was purified by flash chromatography (10→20 vol% EtOAc in petroleum benzine) to afford title compound **S12** (1.95 g, 80%) as a colourless oil.

^{1}H NMR (500 MHz, $CDCl_3$, mixture of rotamers) δ 7.81 – 7.23 (m, 18H), 5.96 – 5.82 (m, 1H), 5.35 – 5.20 (m, 2H), 5.03 (dd, *J* = 12.4, 3.7 Hz, 1H), 4.66 – 4.53 (m, 2H), 4.49 (dd, *J* = 10.6, 7.0 Hz, 0.6H), 4.45 – 4.36 (m, 1.4H), 4.31 (t, *J* = 7.1 Hz, 0.6H), 4.22 (t, *J* = 6.2 Hz, 0.4H), 3.58 – 3.35 (m, 2H), 2.88 (s, 1.8H), 2.85 (s, 1.2H), 2.18 – 2.09 (m, 0.6H), 2.08 – 1.99 (m, 0.4H), 1.71 – 1.46 (m, 2H), 1.07 (s, 5.4H), 1.05 (s, 3.6H), 0.93 (d, *J* = 6.4 Hz, 1.8H), 0.72 (d, *J* = 6.3 Hz, 1.2H). **^{13}C NMR** (125 MHz, $CDCl_3$, mixture of rotamers) δ 171.8, 171.5, 157.1, 156.5, 144.1, 144.0, 143.9, 141.3, 135.6, 135.6, 135.5, 135.2, 134.8, 133.7, 133.7, 133.6, 133.6, 131.8, 131.7, 129.6, 127.7, 127.7, 127.7, 127.6, 127.1, 127.0, 127.0, 125.1, 125.0, 124.7, 120.0, 119.9, 119.9, 118.5, 118.4, 68.9, 68.8, 67.7, 67.5, 65.6, 56.0, 55.9, 53.4, 47.3, 47.2, 32.6, 32.3, 32.2, 31.9, 30.0, 29.8, 26.9, 26.8, 19.2, 19.2, 15.5, 15.2. **LRMS** (+ESI): Mass calculated for $C_{41}H_{47}NO_5Si$: 684.3[M+H]$^+$. Mass found: *m/z* = 684.2 [M+Na]$^+$. **HRMS** (+ESI): Mass calculated for $C_{41}H_{47}NO_5Si$: 684.3122 [M+Na]$^+$. Mass found: 684.3136 [M+Na]$^+$. **IR** v_{max} (ATR) 2956, 2933, 2858, 1742, 1703, 1450, 1319, 1111, 740 cm^{-1}. **[α]$_D$** = -8.5° (*c* 0.96, $CHCl_3$).

*Synthesis of (2S,4S)-2-((((9H-fluoren-9-yl)methoxy)carbonyl)(methyl)amino)-5-((tert-butyldiphenylsilyl)oxy)-4-methylpentanoic acid (**S13**)*

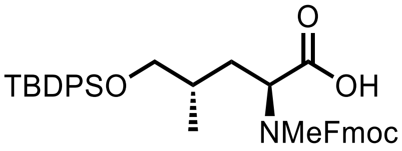

To a solution of **S12** (950 mg, 2.24 mmol) in THF (22 mL) was added *tetrakis*(triphenylphosphine)palladium(0) (26 mg, 0.022 mmol, 1 mol.%) at rt. The mixture was stirred for 10 min, after which phenylsilane (550 mL, 4.5 mmol) was added and stirring continued at room temperature for a further 2 h. The reaction was quenched with 1 M HCl (5 mL) and the aqueous layer was extracted with EtOAc (3 × 50 ml). The combined organic layers were washed with water (2 × 15 mL) and brine (15 mL), dried over $MgSO_4$, filtered and the solvent removed *in vacuo*. The crude residue was purified by flash chromatography (0→10 vol% MeOH in CH_2Cl_2) to afford title compound **S13** as an off-white foam (829 mg, 97%).

^{1}H NMR (500 MHz, $CDCl_3$, mixture of rotamers) δ 7.80 – 7.21 (m, 18H), 5.01 (dd, *J* = 12.2, 3.9 Hz, 0.6H), 4.60 (dd, *J* = 10.6, 5.9 Hz, 0.4H), 4.55 (dd, *J* = 12.2, 3.7 Hz, 0.4H), 4.50 (dd, *J* = 10.6, 7.0 Hz, 0.6H), 4.46 – 4.41 (m, 1H), 4.30 (t, *J* = 7.0 Hz, 0.6H), 4.22 (t, *J* = 5.9 Hz, 0.4H), 3.55 (dd, *J* = 9.9, 5.2 Hz, 0.6H), 3.47 (dd, *J* = 9.8, 5.1 Hz, 0.4H), 3.44 (dd, *J* = 9.9, 7.3 Hz, 0.6H), 3.35 (dd, *J* = 9.9, 7.0 Hz, 0.4H), 2.87 (s, 1.8H), 2.85 (s, 1.2H), 2.17 (ddd, *J* = 14.4, 12.2, 2.6 Hz, 0.6H), 2.07 – 1.99 (m, 0.4H), 1.71 – 1.44 (m, 2H), 1.07 (s, 5.4H), 1.04 (s, 3.6H), 0.92 (d, *J* = 6.5 Hz, 1.8H), 0.69 (d, *J* = 6.3 Hz, 1.2H). **^{13}C NMR** (126 MHz, $CDCl_3$, mixture of rotamers) δ 177.1, 176.6, 157.4, 156.6, 144.2, 144.1, 144.0, 141.5, 141.5, 141.5, 135.7, 135.7, 135.7, 133.8, 133.8, 133.7,

133.7, 132.4, 132.3, 132.3, 129.9, 129.8, 128.8, 128.7, 127.9, 127.8, 127.8, 127.8, 127.2, 127.2, 125.2, 125.2, 124.9, 124.8, 120.1, 120.1, 120.1, 69.0, 68.9, 67.9, 67.7, 56.2, 55.8, 47.4, 47.4, 32.7, 32.5, 32.2, 31.9, 30.3, 30.2, 27.0, 27.0, 20.8, 19.4, 19.4, 15.6, 15.3. **LRMS** (+ESI): Mass calculated for $C_{38}H_{43}NO_5Si$: 644.3 $[M+Na]^+$. Mass found: *m/z* = 644.3 $[M+Na]^+$. **HRMS** (+ESI): Mass calculated for $C_{38}H_{43}NO_5Si$: 644.2803 $[M+Na]^+$. Mass found: 644.2818 $[M+Na]^+$. **IR** (ATR): ν_{max} 2958, 2858, 1704, 1450, 1428, 1392, 1318, 1162, 1106 cm^{-1}. **[α]$_D$** = -6.8° (*c* 1.12, $CHCl_3$).

*Synthesis of alcohol **S14***

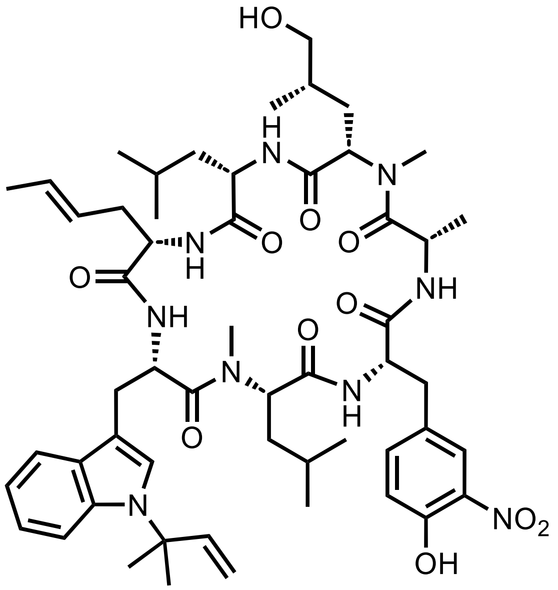

Fmoc-3-NO_2Tyr-OH (144 mg, 0.32 mmol) and *i*-Pr_2NEt (110 µL, 0.6 mmol) were dissolved in CH_2Cl_2 (1 mL) and shaken for 16 h at room temperature with 2-CTC 1% DVB (140 mg, 100 µmol, 0.70 mmol g^{-1}) in a fritted syringe. The loading mixture was discharged, and the resin was washed with CH_2Cl_2 (5 × 3 mL) and DMF (5 × 3 mL). The loaded resin was then capped by shaking for 20 min in a 17:2:1 v/v/v solution of CH_2Cl_2, MeOH, and *i*-Pr_2NEt (3 mL). Fmoc-*N*-Me-Leu-OH (145 mg, 0.40 mmol) was coupled with PyAOP (210 mg, 0.40 mmol), HOAt (110 mg, 0.80 mmol), and NMM (80 µL, 0.8 mmol) in DMF (1.0 mL). The resin was subject to Fmoc-deprotection and Fmoc-*N'*-*tert*-prenyl-L-tryptophan (**S3**) (59 mg, 0.12 mmol) was coupled with PyAOP (63 mg, 120 µmol), HOAt (33 mg, 240 µmol), and NMM (25 µL, 240 µmol) in DMF (1.0 mL) followed by a second coupling of Fmoc-*N'*-*tert*-prenyl-L-tryptophan (**S3**) (32 mg, 64 µmol), PyAOP (33 mg, 64 µmol), HOAt (17 mg, 128 µmol), and NMM (14 µL, 128 µmol) in DMF (1.0 mL). Resin-bound linear peptide was subject to Fmoc-deprotection followed by a 16 h coupling to compound **S5** (37 mg, 104 µmol), PyAOP (54 mg, 104 µmol), HOAt (28 mg, 208 µmol), and NMM (25 µL, 208 µmol) in DMF (1.0 mL). Fmoc-Leu-OH (142 mg, 0.40 mmol) was then coupled with PyAOP (210 mg, 0.40 mmol), HOAt (110 mg, 0.80 mmol), and NMM (80 µL, 0.8 mmol) in DMF (1.0 mL) followed by Fmoc-deprotection and a 12 h coupling of compound **S13** (100 mg, 0.16 mmol), PyAOP (83 mg, 0.16 mmol), HOAt (44 mg, 0.32 mmol), and NMM (35 µL, 320 µmol) in DMF (1.0 mL). Finally, Fmoc-Ala-OH (124 mg, 0.40 mmol) was double coupled with

PyAOP (210 mg, 0.40 mmol), HOAt (110 mg, 0.80 mmol), and NMM (80 µL, 0.8 mmol) in DMF (1.0 mL). Resin-bound linear peptide was shaken in 30 vol.% HFIP in CH_2Cl_2 (2 mL, 2 × 2 h) and washed with CH_2Cl_2 (5 mL). The expelled solutions were combined and concentrated under a stream of nitrogen to afford a crude solid which was subject to cyclisation without purification. Crude peptide was dissolved in CH_2Cl_2 (250 µL) and cooled to 0ºC. A 250 µL aliquot was taken from a stock solution of PyBOP (29 mg, 55 µmol) in CH_2Cl_2 (2.5 mL) and added to the peptide solution. The mixture was stirred at 0 ºC for 2 min before a 100 µL aliquot from a stock solution of *i*-Pr_2NEt (44 µL, 250 µmol) in CH_2Cl_2 (1.0 mL) was added. The mixture was warmed to room temperature and stirred for 16 h after which it was quenched with saturated aqueous ammonium chloride (3 mL) and extracted into EtOAc (3 × 5 mL). The combined organic layers were washed with water (2 mL) and brine (2 mL) and concentrated *in vacuo* to afford protected cyclic peptide which was used without further purification. Crude residue was dissolved in anhydrous THF (4 mL) and cooled to 0 ºC. Tetra-*n*-butylammonium fluoride (400 µL, 1.0 M in THF, 0.4 mmol) was added dropwise, after which the mixture was warmed to room temperature and stirred for 2 h. The reaction was quenched by the dropwise addition of saturated aqueous ammonium chloride (2 mL) and extracted with EtOAc (3 × 10 mL). The combined organic extracts were washed with brine (10 mL), dried over $MgSO_4$, filtered, and concentrated *in vacuo.* The crude residue was purified by RP-HPLC using Waters Sunfire C18 OBD 19×150 mm column (50-100 vol% MeCN in H_2O with 0.1 vol% TFA over 40 min, 14 mL min^{-1}) and lyophilised to afford pure title compound **S14** as a fluffy yellow solid (5.60 mg, 5.5 µmol, 6.8% based on 80 µmol calculated resin loading).

^{1}H NMR (500 MHz, MeOD) δ 8.94 (s, 1H), 7.78 (d, *J* = 2.2 Hz, 1H), 7.53 (m, 1H), 7.51 (m, 1H), 7.41 (dd, *J* = 8.5,2.2 Hz, 1H), 7.13 (s, 1H), 7.08 (m, 1H), 7.06 (m, 1H), 7.03 (m, 1H), 6.14 (m, 1H), 5.5 (m, 1H), 5.2 (m, 2H), 5.17 (m, 1H), 5.06 (m, 1H), 4.91 (dd, *J* = 11.1,4.2 Hz, 1H), 4.83 (m, 1H), 4.66 (m, 1H), 4.60 (m, 1H), 4.6 (m, 1H), 4.58 (m, 1H), 3.46 (dd, *J* = 10.8, 5.4 Hz, 1H), 3.42 (dd, *J* = 13.6, 11.4 Hz, 1H), 3.33 (dd, *J* = 10.2, 7.5 Hz, 1H), 3.17 (dd, *J* = 13.3, 4.5 Hz, 1H), 3.04 (dd, *J* = 13.0, 9.8 Hz, 1H), 2.78 (m, 1H), 2.74 (m, 1H), 2.72 (m, 1H), 2.67 (m, 3H), 2.49 (m, 1H), 1.83 (m, 2H), 1.75 (m, 2H), 1.73 (m, 1H), 1.72 (s, 6H), 1.60 (m, 2H), 1.57 (m, 3H), 1.48 (m, 1H), 1.24 (m, 3H), 1.08 (m, 1H), 0.97 (m, 3H), 0.94 m, 3H), 0.94 (m, 3H), 0.38 (d, *J* = 6.5 Hz, m, 3H), 0.18 (d, *J* = 6.5 Hz, m, 3H). **^{13}C NMR** (125 MHz, MeOD) δ 174.9, 173.7, 173.1, 171.9, 171.4 , 16.9, 16.9, 154.3 , 145, 138.8, 136.7, 135.3, 131, 130.1, 129.4, 126.3, 125, 124.7, 121.8, 121.3, 119.8, 119.1, 114.9, 113.6, 108.6, 68, 68, 59.9, 59.6, 59.5, 55.0, 54.9, 53.1, 51.6, 46.5, 44.3, 38.1, 37.7, 37.7, 35.8, 35.8, 33.4, 33.1, 29.4, 29.3, 28.2, 28.2, 28, 28, 25.7, 25.2, 23.1, 22.9, 21.4, 20.7, 18.2, 16.9, 16.9. **LRMS** (ESI+): Mass calculated for $C_{54}H_{77}N_9O_{11}$: 1028.3 $[M+H]^+$. Mass found: *m/z* = 1028.7 $[M+H]^+$. **HRMS** (ESI+): Mass calculated for $C_{54}H_{77}N_9O_{11}$: 1050.5635 $[M+Na]^+$. Mass found: *m/z* = 1050.5626 $[M+Na]^+$.

*Synthesis of ilamycin E (**2**).*

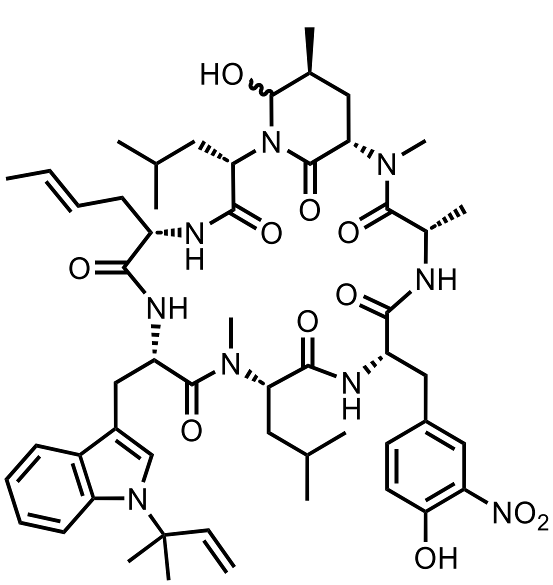

S14 (2.1 mg, 2.0 µmol) was dissolved in CH_2Cl_2 (2.1 mL) under an air atmosphere and cooled to 0 °C. The solution was stirred vigorously and Dess-Martin periodinane (6 mg, 15 µmol) was added. The mixture was warmed to room temperature and stirred at this temperature for 2 h. The reaction mixture was quenched with saturated aqueous Na_2SO_3 (3 mL) and extracted with EtOAc (3 × 10 mL). The combined organic extracts were washed with saturated aqueous Na_2SO_3 (2 mL), brine (2 mL), dried over Na_2SO_4, filtered, and concentrated *in vacuo*. The crude aldehyde was dissolved in MeOH (1.0 mL), K_2CO_3 (1.4 mg, 10 µmol) was added, and the reaction mixture was stirred at rt for 16 h. Volatiles were removed under a stream of nitrogen, affording a crude residue which was purified by RP-HPLC using Waters Sunfire C18 column 5 µm, 10×250 mm (50-100 vol% MeCN in H_2O with 0.1 vol% TFA over 40 min, 4 mL min^{-1}) and lyophilised to afford ilamycin E (**2**) as a pale-yellow solid (0.85 mg, 0.83 µmol, 42%).

^{1}H NMR (600 MHz, MeOD) δ 7.79 – 7.70 (m, 1H), 7.52 (dd, *J* = 7.8, 1.4 Hz, 1H), 7.49 (d, *J* = 8.2 Hz, 1H), 7.33 – 7.26 (m, 1H), 7.18 – 7.11 (m, 1H), 6.97 (d, *J* = 8.8 Hz, 3H), 6.17 – 6.09 (m, 1H), 5.66 – 5.60 (m, 1H), 5.59 – 5.53 (m, 1H), 5.24 (dd, *J* = 11.7, 5.0 Hz, 3H), 4.80 – 4.75 (m, 2H), 4.64 (dd, *J* = 10.4, 6.3 Hz, 1H), 4.59 – 4.55 (m, 1H), 4.30 (dd, *J* = 10.8, 3.8 Hz, 1H), 3.81 – 3.76 (m, 1H), 3.25 – 3.17 (m, 5H), 3.03 (dd, *J* = 14.3, 6.3 Hz, 1H), 2.87 – 2.75 (m, 2H), 2.63 – 2.57 (m, 1H), 2.39 – 2.32 (m, 3H), 2.31 – 2.29 (m, 1H), 2.28 – 2.24 (m, 1H), 1.97 – 1.88 (m, 2H), 1.88 – 1.83 (m, 1H), 1.72 (s, 3H), 1.71 (s, 3H), 1.68 – 1.64 (m, 3H), 1.56 – 1.51 (m, 1H), 1.41 – 1.37 (m, 3H), 1.27 (d, *J* = 6.7 Hz, 3H), 1.09 (d, *J* = 6.7 Hz, 3H), 1.03 – 1.01 (m, 3H), 1.00 – 0.96 (m, 1H), 0.91 (d, *J* = 6.6 Hz, 3H), 0.44 (d, *J* = 6.7 Hz, 3H), 0.13 (d, *J* = 6.8 Hz, 3H), -0.40 – -0.46 (m, 1H). **^{13}C NMR** (150 MHz, MeOD) δ 173.9, 173.1, 172.9, 172.2, 171.6, 171.6, 169.5, 153.4, 145.2, 138.0, 136.6, 136.0, 134.3, 130.2, 129.1, 126.7, 126.4, 124.8, 122.1, 121.9, 119.9, 119.3, 114.8, 113.4, 108.4, 78.9, 62.7, 59.6, 59.1, 56.5, 54.6, 54.0, 51.4,

47.5, 38.1, 37.7, 37.2, 35.5, 34.9, 34, 29, 28.6, 28.2, 28.2, 26.5, 25.6, 25.2, 23.5, 22.7, 21.0, 20.5, 18.1, 17.4, 16.8. **LRMS** (ESI+): Mass calculated for $C_{54}H_{75}N_9O_{11}$: 1048.5 $[M+Na]^+$. Mass found: *m/z* = 1048.5 $[M+Na]^+$; **HRMS:** (+ESI) Calc. for $C_{54}H_{77}N_9O_{11}$: 1048.5478 $[M+Na]^+$, Found: 1048.5467 $[M+Na]^+$; **Analytical HPLC:** R_t 28.1 min (0-100% MeCN (0.1% TFA) in H_2O (0.1% TFA) over 30 min, λ = 214 nm). These data are in agreement with those reported for the isolated natural product.[11]

*Analytical RP-HPLC trace (λ = 214 nm) of ilamycin E (**2**)*

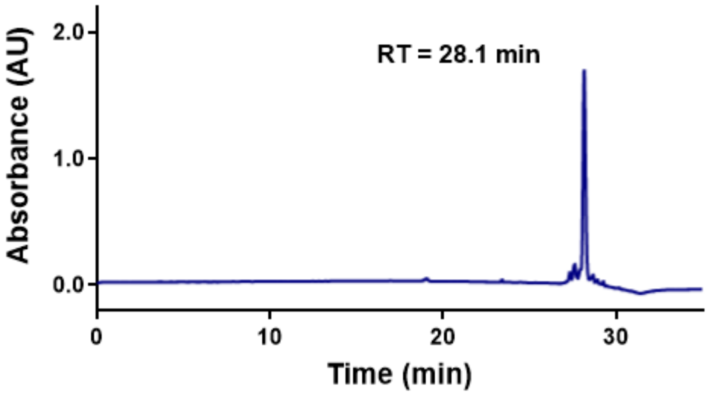

*High-resolution mass spectrum (+ESI) of ilamycin E (**2**)*

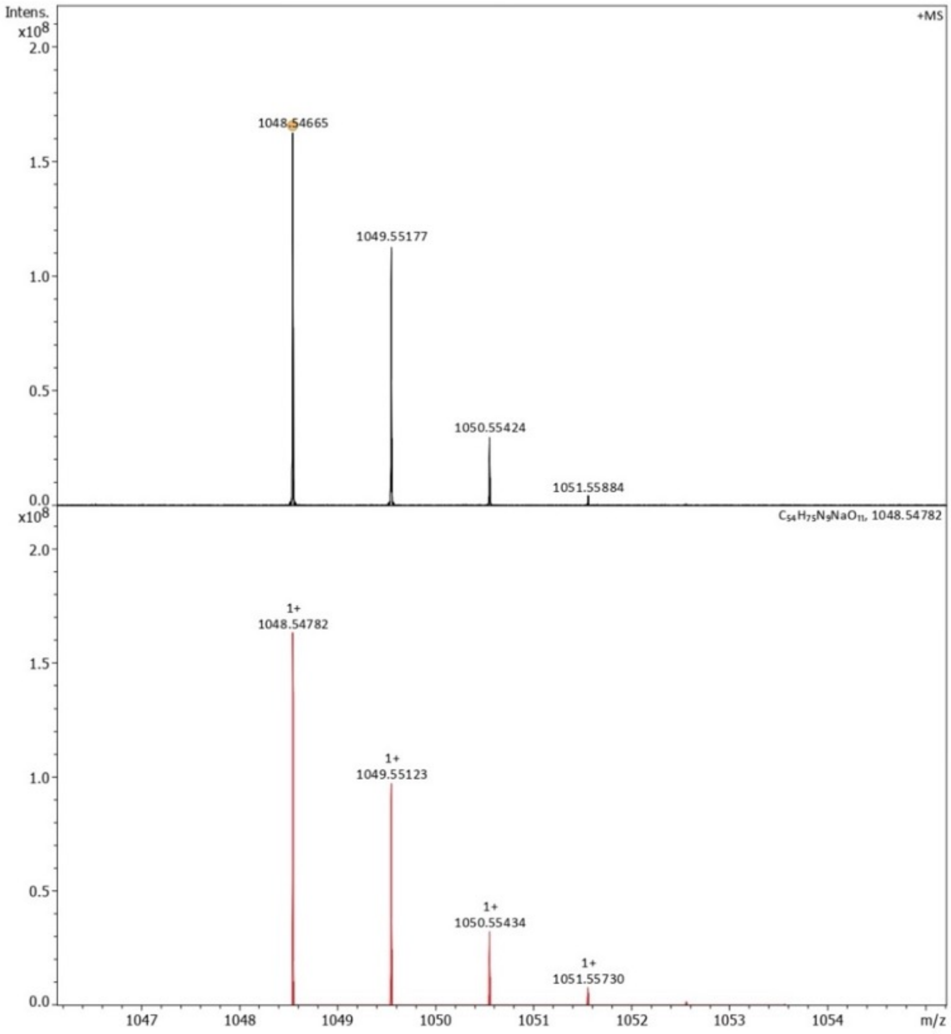

^1H NMR spectrum (600 MHz) of ilamycin E (**2**) (600 MHz, CD_3OD)

	Parameter	Value
1	Data File Name	Z:/ Max/ Rufomycins NMR/ 220325_mbed2504_3002 0546392_IlaEfinal/ 10 (1D)/ fid
2	Title	220325_mbed2504_3002 0546392_IlaEfinal/ 10 (1D)
3	Comment	MB-IlaE
4	Origin	Bruker BioSpin GmbH
5	Owner	chemistry
6	Site	
7	Instrument	spect
8	Author	
9	Solvent	MeOD
10	Temperature	299.9
11	Pulse Sequence	zg30
12	Experiment	1D
13	Probe	Z44896_0030 (CP TCI 600S3 H-C/ N-D-05 Z)
14	Number of Scans	128
15	Receiver Gain	129.7
16	Relaxation Delay	1.0000
17	Pulse Width	8.5000
18	Presaturation Frequency	
19	Acquisition Time	2.7263
20	Acquisition Date	2022-03-25T19:49:47
21	Modification Date	2022-03-25T19:49:48
22	Class	
23	Spectrometer Frequency	600.13
24	Spectral Width	12019.2
25	Lowest Frequency	-2315.4
26	Nucleus	1H
27	Acquired Size	32768
28	Spectral Size	131072
29	Digital Resolution	0.09

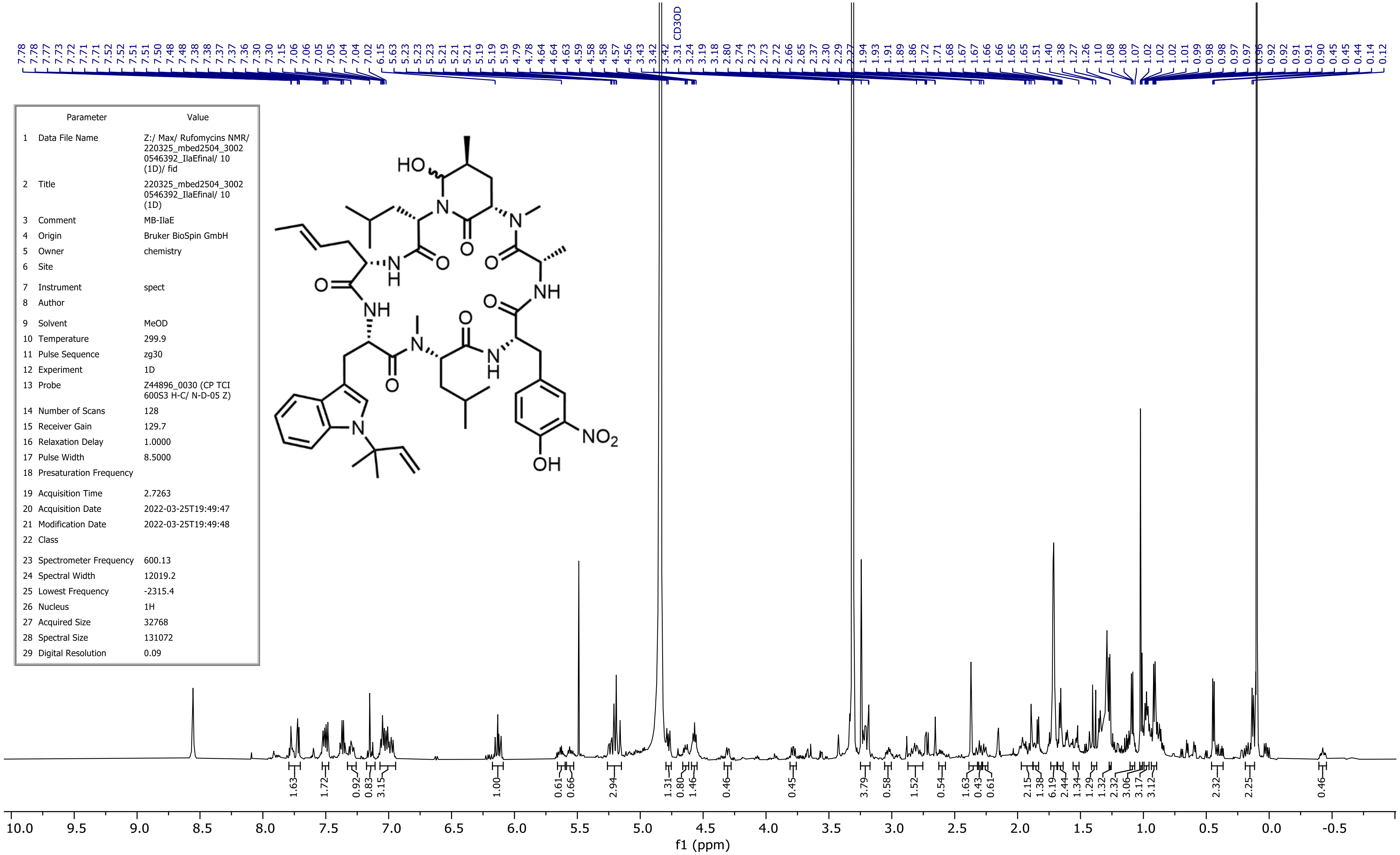

*COSY spectrum of ilamycin E (**2**) (600, 150 MHz, CD_3OD)*

	Parameter	Value
1	Data File Name	Z:/ Max/ Rufomycins NMR/ 220325_mbed2504_30020546392_IlaEfinal/ 2 (COSY)/ ser
2	Title	220325_mbed2504_30020546392_IlaEfinal/ 2 (COSY)
3	Comment	MB-IlaE
4	Origin	Bruker BioSpin GmbH
5	Owner	chemistry
6	Site	
7	Instrument	spect
8	Author	
9	Solvent	MeOD
10	Temperature	300.0
11	Pulse Sequence	cosygpmfqf
12	Experiment	COSY
13	Probe	Z44896_0030 (CP TCI 600S3 H-C/ N-D-05 Z)
14	Number of Scans	24
15	Receiver Gain	884.7
16	Relaxation Delay	2.0000
17	Pulse Width	8.5000
18	Presaturation Frequency	
19	Acquisition Time	0.3408
20	Acquisition Date	2022-03-25T20:04:49
21	Modification Date	2022-03-25T22:04:39
22	Class	
23	Spectrometer Frequency	(600.13,)
24	Spectral Width	(6009.6,)
25	Lowest Frequency	(-16.6,)
26	Nucleus	(1H,)
27	Acquired Size	(2048, 128)
28	Spectral Size	(2048,)
29	Digital Resolution	(2.93,)

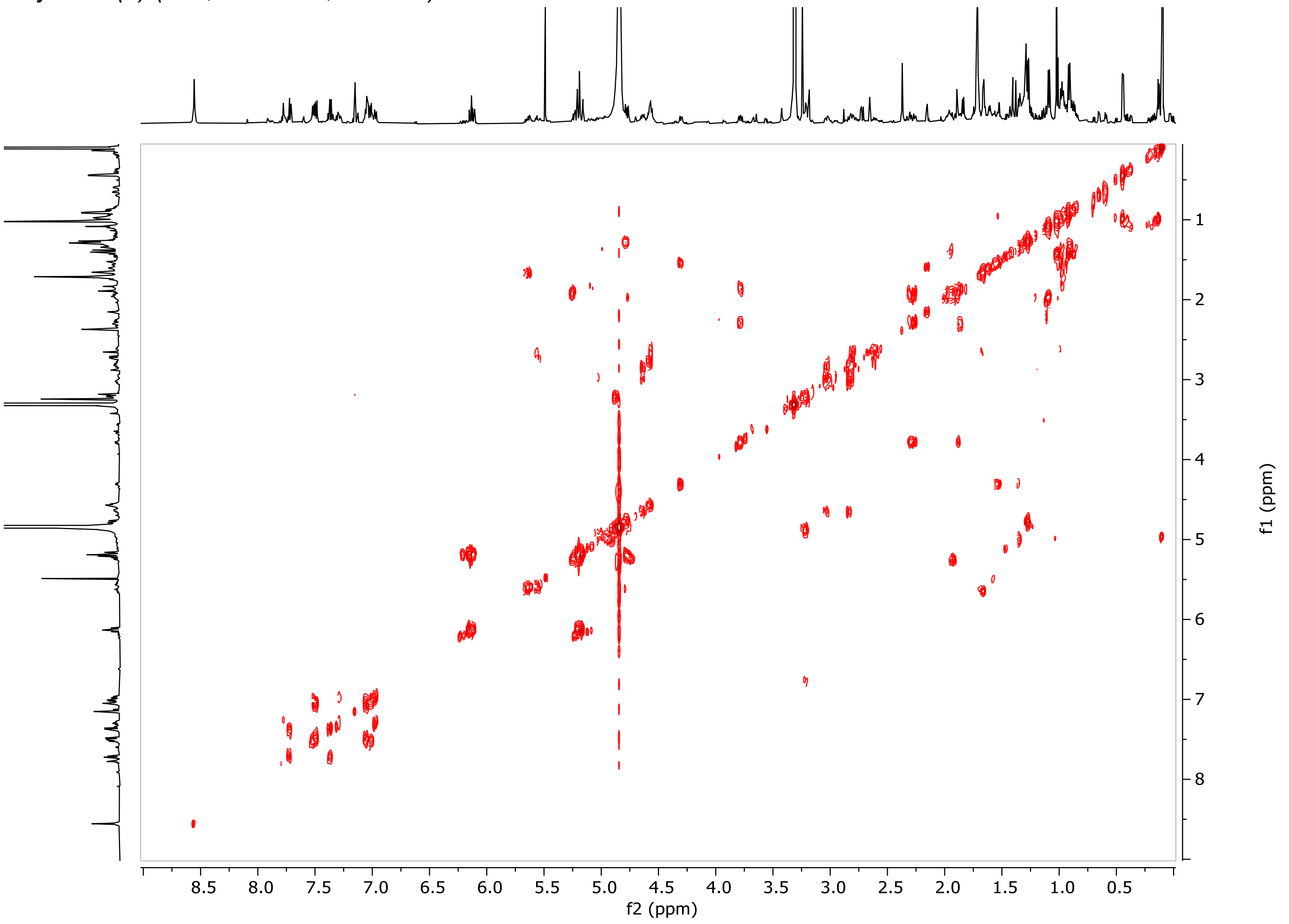

*HSQC spectrum of ilamycin E (**2**) (600, 150 MHz, CD_3OD)*

	Parameter	Value
1	Data File Name	Z:/ Max/ Rufomycins NMR/ 220325_mbed2504_30 020546392_IlaEfinal/ 6 (HSQC)/ ser
2	Title	220325_mbed2504_30 020546392_IlaEfinal/ 6 (HSQC)
3	Comment	MB-IlaE
4	Origin	Bruker BioSpin GmbH
5	Owner	chemistry
6	Site	
7	Instrument	spect
8	Author	
9	Solvent	MeOD
10	Temperature	298.0
11	Pulse Sequence	hsqcedetgp
12	Experiment	HSQC-EDITED
13	Probe	Z44896_0030 (CP TCI 600S3 H-C/ N-D-05 Z)
14	Number of Scans	60
15	Receiver Gain	2050.0
16	Relaxation Delay	1.5000
17	Pulse Width	8.5000
18	Presaturation Frequency	
19	Acquisition Time	0.2171
20	Acquisition Date	2022-03-26T10:09:04
21	Modification Date	2022-03-26T15:45:54
22	Class	
23	Spectrometer Frequency	(600.13,)
24	Spectral Width	(4717.0,)
25	Lowest Frequency	(47.1,)
26	Nucleus	(1H,)
27	Acquired Size	(1024, 195)
28	Spectral Size	(1024,)
29	Digital Resolution	(4.61,)

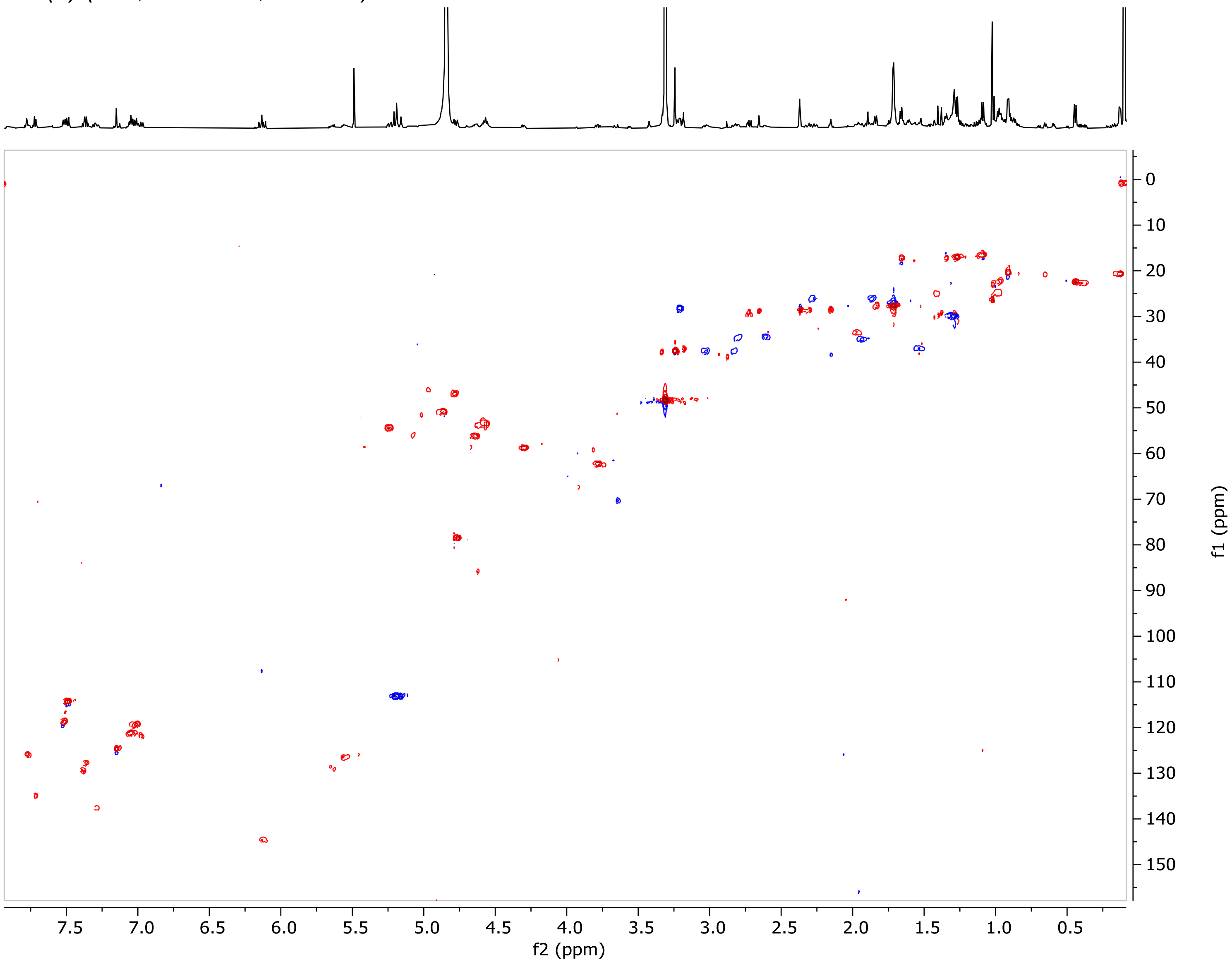

*HMBC spectrum of ilamycin E (**2**) (600, 150 MHz, CD_3OD)*

	Parameter	Value
1	Data File Name	Z:/ Max/ Rufomycins NMR/ 220325_mbed2504_30020546392_IlaEfinal/ 7 (HMBC)/ ser
2	Title	220325_mbed2504_30020546392_IlaEfinal/ 7 (HMBC)
3	Comment	MB-IlaE
4	Origin	Bruker BioSpin GmbH
5	Owner	chemistry
6	Site	
7	Instrument	spect
8	Author	
9	Solvent	MeOD
10	Temperature	298.1
11	Pulse Sequence	hmbcgplpndqf
12	Experiment	HMBC
13	Probe	Z44896_0030 (CP TCI 600S3 H-C/ N-D-05 Z)
14	Number of Scans	64
15	Receiver Gain	2050.0
16	Relaxation Delay	1.5000
17	Pulse Width	8.5000
18	Presaturation Frequency	
19	Acquisition Time	0.2171
20	Acquisition Date	2022-03-26T15:48:34
21	Modification Date	2022-03-27T08:01:35
22	Class	
23	Spectrometer Frequency	(600.13,)
24	Spectral Width	(4717.0,)
25	Lowest Frequency	(45.2,)
26	Nucleus	(1H,)
27	Acquired Size	(1024, 512)
28	Spectral Size	(1024,)
29	Digital Resolution	(4.61,)

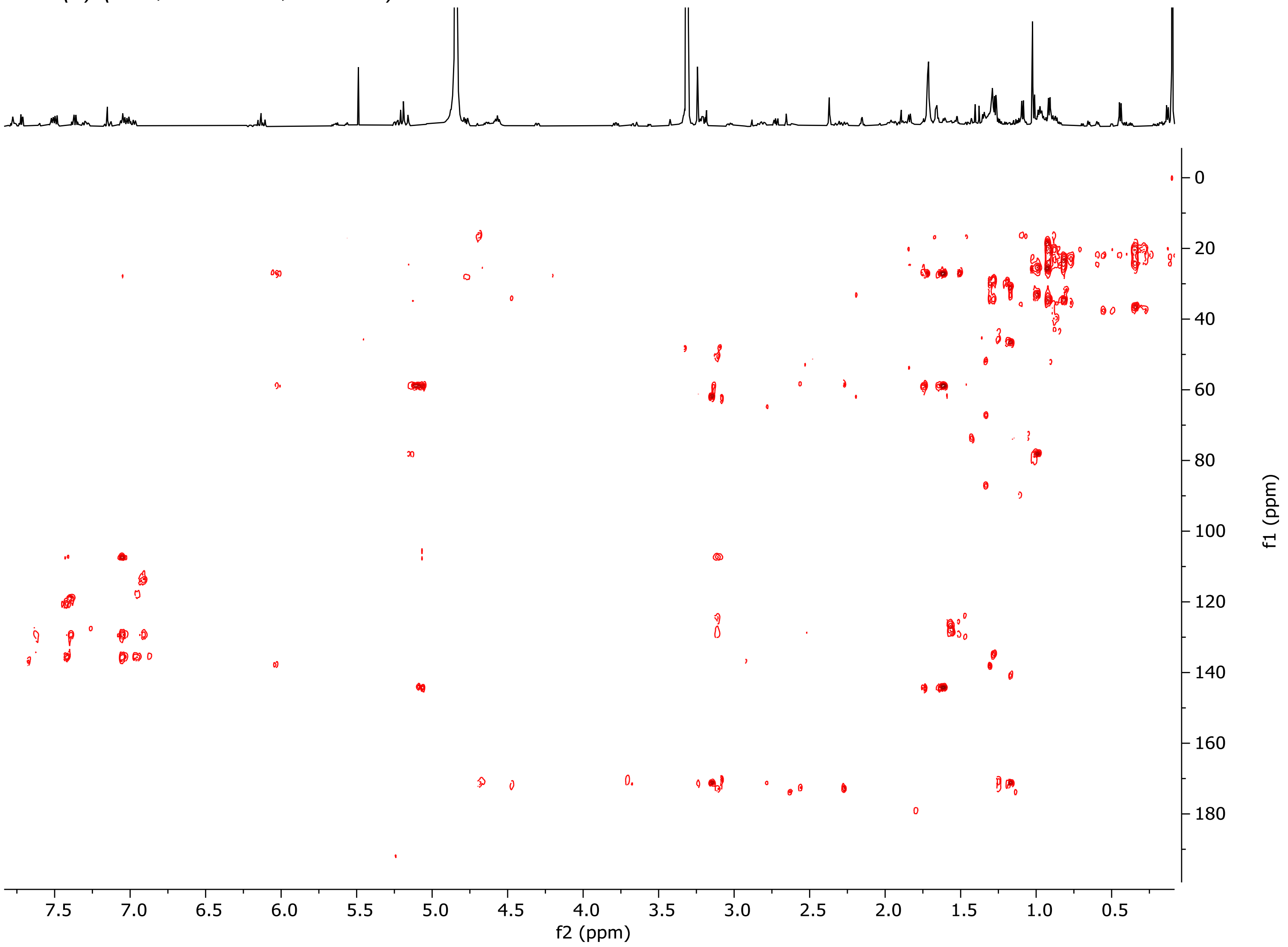

References

1. Wolf, N.M., *et al.* Structure of the N-terminal domain of ClpC1 in complex with the antituberculosis natural product ecumicin reveals unique binding interactions. *Acta Crystallogr D Struct Biol* **76**, 458-471 (2020).
2. Hoi, D.M., *et al.* Clp-targeting BacPROTACs impair mycobacterial proteostasis and survival. *Cell* **186**, 2176-2192 e2122 (2023).
3. Wolf, N.M., *et al.* High-Resolution Structure of ClpC1-Rufomycin and Ligand Binding Studies Provide a Framework to Design and Optimize Anti-Tuberculosis Leads. *ACS Infect Dis* **5**, 829-840 (2019).
4. Bedding, M.J., *et al.* Modular Total Synthesis and Antimycobacterial Activity of Rufomycins. *Org Lett* **26**, 10993-10998 (2024).
5. Ma, J., *et al.* Biosynthesis of ilamycins featuring unusual building blocks and engineered production of enhanced anti-tuberculosis agents. *Nature Communications* **8**(2017).
6. Supek, F., Bosnjak, M., Skunca, N. & Smuc, T. REVIGO summarizes and visualizes long lists of gene ontology terms. *PLoS One* **6**, e21800 (2011).
7. Speckmeier, E., Klimkait, M. & Zeitler, K. Unlocking the Potential of Phenacyl Protecting Groups: CO(2)-Based Formation and Photocatalytic Release of Caged Amines. *J Org Chem* **83**, 3738-3745 (2018).
8. Luzung, M.R., Lewis, C.A. & Baran, P.S. Direct, chemoselective N-tert-prenylation of indoles by C-H functionalization. *Angew Chem Int Ed Engl* **48**, 7025-7029 (2009).
9. Baran, P.S., Guerrero, C.A. & Corey, E.J. Short, enantioselective total synthesis of okaramine N. *J Am Chem Soc* **125**, 5628-5629 (2003).
10. Cergol, K.M., Thompson, R.E., Malins, L.R., Turner, P. & Payne, R.J. One-pot peptide ligation-desulfurization at glutamate. *Org Lett* **16**, 290-293 (2014).
11. Ma, J., *et al.* Biosynthesis of ilamycins featuring unusual building blocks and engineered production of enhanced anti-tuberculosis agents. *Nat Commun* **8**, 391 (2017).
