# Supplementary material for: ClpC1-targeting peptide natural products differentially dysregulate the proteome of Mycobacterium tuberculosis
Source: Nat Commun. 2026 Jan 29;17:1725. doi: 10.1038/s41467-026-68423-2 (PMC12913811; doi:10.1038/s41467-026-68423-2)
Supplement: Supplementary file 2 — Description of Additional Supplementary Files [file 41467_2026_68423_MOESM2_ESM.pdf]

Supplementary Data 1: Raw LC-MS/MS proteomic data and statistics following NRP treatment

Supplementary Data 2: Gene ontology enrichment analysis of differentially abundant proteins following NRP treatment

Supplementary Data 3: Termini disorder of Mtb proteins

Supplementary Data 4: Raw LC-MS/MS proteomic data and statistics following BTZ (**4**) treatment

Supplementary Data 5: RNA sequencing data and statistics following Ecu* (**1**) treatment
