# Supplementary material for: ClpC1-targeting peptide natural products differentially dysregulate the proteome of Mycobacterium tuberculosis
Source: Nat Commun. 2026 Jan 29;17:1725. doi: 10.1038/s41467-026-68423-2 (PMC12913811; doi:10.1038/s41467-026-68423-2)
Supplement: Supplementary file 8 — Reporting Summary [file 41467_2026_68423_MOESM8_ESM.pdf]

nature portfolio

Corresponding author(s): Richard J. Payne, Mark Larance, Warwick J. Britton

Last updated by author(s): 20/10/2025

Reporting Summary

Nature Portfolio wishes to improve the reproducibility of the work that we publish. This form provides structure for consistency and transparency in reporting. For further information on Nature Portfolio policies, see our Editorial Policies and the Editorial Policy Checklist.

Statistics

For all statistical analyses, confirm that the following items are present in the figure legend, table legend, main text, or Methods section.

n/a	Confirmed	
☐	☒	The exact sample size (*n*) for each experimental group/condition, given as a discrete number and unit of measurement
☐	☒	A statement on whether measurements were taken from distinct samples or whether the same sample was measured repeatedly
☐	☒	The statistical test(s) used AND whether they are one- or two-sided<br>*Only common tests should be described solely by name; describe more complex techniques in the Methods section.*
☒	☐	A description of all covariates tested
☐	☒	A description of any assumptions or corrections, such as tests of normality and adjustment for multiple comparisons
☐	☒	A full description of the statistical parameters including central tendency (e.g. means) or other basic estimates (e.g. regression coefficient) AND variation (e.g. standard deviation) or associated estimates of uncertainty (e.g. confidence intervals)
☐	☒	For null hypothesis testing, the test statistic (e.g. *F*, *t*, *r*) with confidence intervals, effect sizes, degrees of freedom and *P* value noted<br>*Give P values as exact values whenever suitable.*
☒	☐	For Bayesian analysis, information on the choice of priors and Markov chain Monte Carlo settings
☒	☐	For hierarchical and complex designs, identification of the appropriate level for tests and full reporting of outcomes
☐	☒	Estimates of effect sizes (e.g. Cohen's *d*, Pearson's *r*), indicating how they were calculated

Our web collection on statistics for biologists contains articles on many of the points above.

Software and code

Policy information about availability of computer code

Data collection	MS: Thermo Fisher VanquishT Neo UHPLC system coupled with an Orbitrap ExplorisTM 480 mass spectrometer, an Orbitrap Fusion Lumos Tribrid mass spectrometer or a Q Exactive Plus Hybrid Quadrupole-Orbitrap mass spectrometer Tune 2.12 (Build 3134), Xcalibur (version 4.4.16.14) and SciexOS (version 3.4.0.19154)<br>Surface plasma resonance: GE Healthcare Biacore™ T200, Biacore Insight Evaluation software (version 6.0)<br>Plate reader: BMG Labtech POLARstar Omega microplate reader<br>Gel imager: ChemiDoc MP BioRad Laboratories<br>Size exclusion chromatography:Thermo Fisher U3000 BioRS UHPLC, Cytiva Akta pure purification system<br>NMR spectroscopy: Bruker Avance DRX300, DPX400, DPX500, or DPX600 spectrometers.<br>Infrared (IR) absorption spectroscopy: Bruker Alpha Spectrometer.<br>Optical rotation: Perkin Elmer Model 341 polarimeter<br>Low resolution mass spectrometry: Bruker amaZon SL mass spectrometer or Shimadzu 2020 (ESI) mass spectrometer.<br>High resolution mass spectrometry: Bruker Apex Qe 7T Fourier Transform Ion Cyclotron Resonance Mass Spectrometer (FTICR) mass spectrometer.<br>UPLC: Waters Acquity H-class.<br>Preparative reversed-phase HPLC: Waters 2535 Multisolvent Delivery System with a Waters 2489 UV/visible detector
Data analysis	General Data Analysis: GraphPad Prism (version 10.3.0), Microsoft Excel (version 16.89.1), Tableau (version 2024.1.2)<br>MS: DIA NN (version 1.8.1), MaxQuant (version 1.6.3.4)<br>Statistical Analysis: R studio (version 4.2.1)

Protein structural analysis: UCSF ChimeraX (version 1.7.1)
Gene ontology analysis: STRINGdb (version 12.0), Revigo (version 1.8.1)
Disorder prediction: MobiDB (version 5.0)
Gel band intensity quantification: ImageJ (version 1. 54p)
analytical SEC: Chromeleon (version 6.8)

For manuscripts utilizing custom algorithms or software that are central to the research but not yet described in published literature, software must be made available to editors and reviewers. We strongly encourage code deposition in a community repository (e.g. GitHub). See the Nature Portfolio guidelines for submitting code & software for further information.

Data

Policy information about availability of data

All manuscripts must include a data availability statement. This statement should provide the following information, where applicable:

- Accession codes, unique identifiers, or web links for publicly available datasets
- A description of any restrictions on data availability
- For clinical datasets or third party data, please ensure that the statement adheres to our policy

All data related to this study are provided within the paper, the supplementary information, supplementary data files and Source data file. Raw and processed LC-MS/MS data files along with the Mycobacterium tuberculosis database used in the DIA NN search have been uploaded to EMBL-EBI PRIDE repository (identifier PXD057335). The raw sequence reads have been deposited in the NCBI SRA under PRJNA1346855. Processed data files (normalized counts and VST) have been uploaded to the GEO NCIB-NIH repository (identifier GSE310713).

Research involving human participants, their data, or biological material

Policy information about studies with human participants or human data. See also policy information about sex, gender (identity/presentation), and sexual orientation and race, ethnicity and racism.

Reporting on sex and gender	n/a
Reporting on race, ethnicity, or other socially relevant groupings	n/a
Population characteristics	n/a
Recruitment	n/a
Ethics oversight	n/a

Note that full information on the approval of the study protocol must also be provided in the manuscript.

Field-specific reporting

Please select the one below that is the best fit for your research. If you are not sure, read the appropriate sections before making your selection.

☒ Life sciences ☐ Behavioural & social sciences ☐ Ecological, evolutionary & environmental sciences

For a reference copy of the document with all sections, see nature.com/documents/nr-reporting-summary-flat.pdf

Life sciences study design

All studies must disclose on these points even when the disclosure is negative.

Sample size	Sample sizes were determined according to standards in the field for equivalent experiments. Data was collected from at least n=3 biological replicates for all experiments as specified in the Methods.
Data exclusions	One Ecu* treated Mycobacterium tuberculosis LC-MS/MS dataset was excluded from further analysis due to a suspected experimental anomaly. The experiment was performed a total of 7 times and 6 were included in statistical analysis.
Replication	All biological replicates were obtained from independent experiments at least in triplicate. Reported results were consistently reproducible across a minimum of 3 experiments.
Randomization	Randomization was not applicable in our study as we were comparing the activity of purified ompounds or purified proteins using well-controlled conditions. To account for the lack of randomization, all experiments were performed with appropriate controls.
Blinding	Blinding was not used as observer bias was expected in this study. A negative control was run in parallel with each biological experiment and measurement readouts were objective.

Reporting for specific materials, systems and methods

We require information from authors about some types of materials, experimental systems and methods used in many studies. Here, indicate whether each material, system or method listed is relevant to your study. If you are not sure if a list item applies to your research, read the appropriate section before selecting a response.

Materials & experimental systems

n/a	Involved in the study
☒	☐ Antibodies
☒	☐ Eukaryotic cell lines
☒	☐ Palaeontology and archaeology
☒	☐ Animals and other organisms
☒	☐ Clinical data
☒	☐ Dual use research of concern
☒	☐ Plants

Methods

n/a	Involved in the study
☒	☐ ChIP-seq
☒	☐ Flow cytometry
☒	☐ MRI-based neuroimaging

Plants

Seed stocks	n/a
Novel plant genotypes	n/a
Authentication	n/a
